# Replicated associations of *FADS1*, *MAD1L1*, and a rare variant at 10q26.13 with bipolar disorder in Chinese population

**DOI:** 10.1038/s41398-018-0337-x

**Published:** 2018-12-07

**Authors:** Lijuan Zhao, Hong Chang, Dong-Sheng Zhou, Jun Cai, Weixing Fan, Wei Tang, Wenxin Tang, Xingxing Li, Weiqing Liu, Fang Liu, Yuanfang He, Yan Bai, Yan Sun, Jiapei Dai, Lingyi Li, Xiao Xiao, Chen Zhang, Ming Li

**Affiliations:** 1 Key Laboratory of Animal Models and Human Disease Mechanisms of the Chinese Academy of Sciences and Yunnan Province, Kunming Institute of Zoology, Kunming, Yunnan China; 2 Kunming College of Life Science, University of Chinese Academy of Sciences, Kunming, Yunnan China; 3 Department of Psychiatry, Ningbo Kangning Hospital, Ningbo, Zhejiang China; 4 Shanghai Mental Health Center, Shanghai Jiao Tong University School of Medicine, Shanghai, China; 5 Jinhua Second Hospital, Jinhua, Zhejiang China; 6 Wenzhou Kangning Hospital, Wenzhou Medical University, Wenzhou, Zhejiang China; 7 Hangzhou Seventh People’s Hospital, Hangzhou, Zhejiang China; 8 Department of Psychiatry, the First Affiliated Hospital of Kunming Medical University, Kunming, Yunnan China; 9 Wuhan Institute for Neuroscience and Neuroengineering, South-Central University for Nationalities, Wuhan, Hubei China; 10 Chinese Brain Bank Center, Wuhan, Hubei China; 11 CAS Center for Excellence in Brain Science and Intelligence Technology, Chinese Academy of Sciences, Shanghai, China

## Abstract

Genetic analyses of psychiatric illnesses, such as bipolar disorder (BPD), have revealed essential information regarding the underlying pathological mechanisms. While such studies in populations of European ancestry have achieved prominent success, understanding the genetic risk factors of these illnesses (especially BPD) in Chinese population remains an urgent task. Given the lack of genome-wide association study (GWAS) of BPD in Chinese population from Mainland China, replicating the previously reported GWAS hits in distinct populations will provide valuable information for future GWAS analysis in Han Chinese. In the present study, we have recruited 1146 BPD cases and 1956 controls from Mainland China for genetic analyses, as well as 65 Han Chinese brain amygdala tissues for mRNA expression analyses. Using this clinical sample, one of the largest Han Chinese BPD samples till now, we have conducted replication analyses of 21 single nucleotide polymorphisms (SNPs) extracted from previous GWAS of distinct populations. Among the 21 tested SNPs, 16 showed the same direction of allelic effects in our samples compared with previous studies; 6 SNPs achieved nominal significance (*p* < 0.05) at one-tailed test, and 2 additional SNPs showed marginal significance (*p* < 0.10). Aside from replicating previously reported BPD risk SNPs, we herein also report several intriguing findings: (1) the SNP rs174576 was associated with BPD in our Chinese sample and in the overall global meta-analysis, and was significantly correlated with *FADS1* mRNA in diverse public RNA-seq datasets as well as our in house collected Chinese amygdala samples; (2) two (partially) independent SNPs in *MAD1L1* were both significantly associated with BPD in our Chinese sample, which was also supported by haplotype analysis; (3) a rare SNP rs78089757 in 10q26.13 region was a genome-wide significant variant for BPD in East Asians, and this SNP was near monomorphic in Europeans. In sum, these results confirmed several significant BPD risk genes. We hope this Chinese BPD case–control sample and the current brain amygdala tissues (with continuous increasing sample size in the near future) will provide helpful resources in elucidating the genetic and molecular basis of BPD in this major world population.

## Introduction

Bipolar disorder (BPD) is a complex mental illness with considerable genetic heritability^1–4^. The neurobiology of BPD is not yet fully understood, but accumulating studies have suggested the essential roles of aberrant synaptic function in specific brain regions relevant to emotion and cognition in BPD pathogenesis^5–7^. In lieu with the putative roles of genetic risk factors in the pathogenesis of BPD, genome-wide association studies (GWAS), the approach widely recognized as effective in identifying genetic risk loci for psychiatric disorders^8–12^, have been performed in European and Japanese populations and have reported a number of BPD susceptibility loci spanning genes including *ANK3*, *CACNA1C*, *NCAN*, and others^13–22^. The largest BPD GWAS so far is performed by the Psychiatric Genomics Consortium Bipolar Disorder (PGC-BP) group. They conducted a meta-analysis of multiple GWAS datasets including 20,352 BPD patients and 31,358 controls in European populations, followed by replication analyses of the significant loci (*p* < 1 ⨯ 10^−4^) in an additional 9412 cases and 137,760 controls^14^. This combined meta-analysis of the GWAS discovery and replication samples (named BPD PGC2 GWAS) yielded 30 leading single nucleotide polymorphisms (SNPs) showing genome-wide significance^14^. Before BPD PGC2 GWAS, another two BPD GWAS of smaller samples (these samples were partially overlapped with that of BPD PGC2 GWAS) recruited from European populations have also found several SNPs showing genome-wide level of statistical significance^15,18^. The progress of BPD genetic studies in Asians have also recently emerged, as a GWAS carried out in Japanese have lately identified additional novel risk variants for this illness, suggesting the potential genetic overlap and heterogeneity of BPD between distinct continental populations^17^. These discoveries motivated further characterization of BPD genetic risk factors in other populations, as well as validation of the identified risk associations to understand the generalizability of current GWAS findings.

For these purposes, we thus sought to explore the unclear genetic basis of BPD in Chinese populations. While Chinese populations make up around one fifth of the total world, the progress of genetic analyses for BPD in Han Chinese has been much slower compared to that in other cohorts. Till now, no genome-wide significant risk variants for BPD in Han Chinese population have been reported^23^, and replication studies of European GWAS loci in Chinese BPD sample have been barely published. We recruited 1146 BPD patients and 1956 control subjects from Mainland China to replicate the associations of the 21 risk variants highlighted in previous BPD GWAS (Table 1). Given the hypothesis that risk variants usually contribute to the illnesses by modulating the expression of nearby genes^24,25^, we have also collected 65 brain amygdala tissues from nonpsychiatric individuals of Han Chinese ancestry, and tested whether the BPD risk SNPs affected expression levels of particular genes.

**Table 1:** Replication analyses of GWAS loci for bipolar disorder in an independent Han Chinese sample (1146 BPD cases and 1956 controls)

CHR	SNP	POS	Nearest Gene	Allele	OR	*p*Value	Ref.	Frequency		Two-tailed *p* value	One-tailed *p* value	OR	95% CIs
								Case	Control				
2	**rs17183814**	166152389	*SCN2A*	G/A	1.142	2.02E−09	14	0.873	0.856	0.0623	**0.0311**	1.153	0.993-1.340
3	rs2302417	52814256	*ITIH3*	T/A	1.076	6.59E−11	14	0.609	0.590	0.144	0.0718	1.083	0.974-1.205
3	**rs3804640**	107793709	*CD47*	A/G	1.065	1.99E−08	14	0.865	0.848	0.0738	**0.0369**	1.147	0.987-1.334
4	rs11724116	162294038	*FSTL5*	C/T	1.088	2.37E−08	14	0.863	0.858	0.571	0.285	1.045	0.898-1.215
6	rs10455979	166995260	*RPS6KA2*	G/C	1.064	4.31E−08	14	0.703	0.715	0.336	0.168	0.944	0.840-1.061
7	**rs4236274**	1896413	*MAD1L1*	A/G	1.149	8.49E−12	15	0.478	0.445	**0.0146**	**0.00731**	1.140	1.026-1.266
7	**rs4332037**	1950809	*MAD1L1*	T/C	1.170	1.91E−09	17	0.115	0.097	**0.0209**	**0.0104**	1.218	1.030-1.441
9	rs12553324	23347865	*ELAVL2*	G/C	1.120	5.87E−09	15	0.498	0.512	0.297	0.148	0.946	0.853-1.050
10	rs10821745*	62136206*	*ANK3*	G/T	1.145	6.76E−09	14	0.238	0.236	0.835	0.418	1.013	0.896-1.145
10	**rs78089757**	127112829	intergenic	A/G	1.410	3.99E−07	17	0.026	0.019	0.0881	**0.0440**	1.358	0.955-1.931
11	**rs174576**	61603510	*FADS1/2*	A/C	1.130	1.34E−10	17	0.457	0.430	**0.0459**	**0.0229**	1.110	1.002-1.230
11	rs7122539	66662731	*RCE1*	G/A	1.067	3.77E−08	14	0.517	0.496	0.113	0.0565	1.086	0.980-1.204
11	rs12575685	70517927	*SHANK2*	A/G	1.073	7.71E−09	14	0.278	0.265	0.285	0.143	1.066	0.948-1.197
11	rs12290811	79083620	*ODZ4*	A/T	1.190	7.81E−11	18	0.062	0.057	0.428	0.214	1.093	0.878-1.360
11	rs329674	133776948	*LOC646543*	A/G	1.220	9.59E−08	17	0.193	0.198	0.678	0.339	0.973	0.855-1.107
12	rs10744560	2387099	*CACNA1C*	T/C	1.076	3.62E−10	14	0.060	0.053	0.287	0.143	1.130	0.902-1.415
15	rs4447398	42904904	*HAUS2*	A/C	1.099	9.37E−09	14	0.270	0.274	0.701	0.350	0.977	0.870-1.099
16	rs11647445	9926966	*GRIN2A*	G/T	1.079	1.08E−10	14	0.076	0.076	0.978	0.489	1.003	0.826-1.217
17	rs76317718	8222777	*ARHGEF15*	G/T	1.190	2.24E−07	17	0.723	0.725	0.848	0.424	0.989	0.882-1.109
17	rs112114764	42201041	*HDAC5*	G/T	1.071	2.45E−08	14	0.802	0.790	0.244	0.122	1.080	0.949-1.229
20	rs6130764	43750410	*STK4*	T/C	1.064	3.25E−08	14	0.859	0.855	0.732	0.366	1.027	0.883-1.194

**Note:**

^*^rs10821745 is a proxy SNP for rs10994318 (*r*^2^ = 0.945 in Chinese and *r*^2^ = 1.000 in Europeans from 1000 Genomes Project), which has been reported in a previous GWAS^14^, and the rs10821745’s position is also shown in Table 1

The frequency and OR are based on the first allele shown in the fifth column

*CHR* chromosome, *SNP* single nucleotide polymorphism, *POS* position, *OR* odds ratio, *Ref.* reference, *CIs* confidence intervals

The significant *p* values (*p* < 0.05) were marked in bold

## Methods

### Chinese BPD case–control sample

With the aim to build a DNA resource pool for investigating the genetic bases of BPD in Han Chinese population, we have initially recruited a total of 1146 BPD cases and 1956 controls of Han Chinese origin from Mainland China. The protocol was approved by the institutional review board of the Kunming Institute of Zoology, Chinese Academy of Sciences and the ethics committees of all participating hospitals and universities. All participants provided written informed consents before any study-related procedures were performed.

The BPD patients were collected from multiple provinces of Mainland China, including mental health centers and psychiatric departments of general hospitals. Each patient was diagnosed as having BPD by a consensus of at least two experienced psychiatrists. Diagnoses were further confirmed with an Extensive Clinical Interview and a Structured Clinical Interview for DSM-IV Axis/Disorders, Patient Version (SCID-P), as previously described^26,27^. Cases were excluded if they had either a pre-existing history of schizophrenia, mental retardation, or drug/alcohol addiction. Subjects with concurrent diagnosis of other mental illnesses were also excluded to minimize potential compounding conditions in our analysis. The control subjects were recruited from Mainland China with their medical and family history information records. Subjects with any history of major neuropsychiatric or neurological disorders (e.g., BPD, depression, schizophrenia, attention deficit hyperactivity disorder, and mental retardation), or with a family history of severe mental illnesses, were excluded from this study.

### SNP selection

A total of 21 SNPs were selected based on the results of four previous BPD GWAS studies in European and Japanese populations^14,15,17,18^. Among them, 13 SNPs (rs17183814, rs2302417, rs3804640, rs11724116, rs10455979, rs10821745, rs7122539, rs12575685, rs10744560, rs4447398, rs11647445, rs112114764, and rs6130764) were selected from the BPD PGC2 GWAS (29,764 cases and 169,118 controls of European ancestry) because of their genome-wide significant associations with BPD^14^. Two genome-wide significant SNPs (rs4236274 and rs12553324) were chosen from Hou et al. GWAS (9784 cases and 30,471 controls of European origin)^15^. One genome-wide risk SNP (rs12290811) was obtained from Muhleisen et al.^18^ GWAS (9747 cases and 14,278 controls of European ancestry), and five additional SNPs (rs4332037, rs78089757, rs174576, rs329674, and rs76317718) were chosen from a previous GWAS study by Ikeda et al.^17^ (2964 cases and 61,887 controls of Japanese origin, and 7481 cases and 9250 controls of European ancestry). In this trans-ethnic analysis performed by Ikeda et al.^17^, two (rs4332037 and rs174576) of the five selected SNPs showed genome-wide significant associations with BPD in both Europeans and Japanese; the other three SNPs (rs78089757, rs329674, and rs76317718), though not highlighted in cross-population analysis, all showed marginal genome-wide significance in Japanese (*p* < 5.00E−07). Given the similar genetic background between Japanese and Han Chinese populations, we also examined whether these three SNPs (rs78089757, rs329674, and rs76317718) were associated with BPD in Han Chinese, and whether the associations could reach genome-wide level of significance when all the East Asian samples were combined. The statistics (*p* values and odds ratios (ORs)) of the 21 SNPs were retrieved from the original studies and summarized in Table 1.

### SNP genotyping

Peripheral venous blood samples were collected and were stored at −80 °C. Genomic DNA was extracted from peripheral blood leukocytes using high-salt extraction procedures according to the manufacturer’s protocol. Each sample was genotyped at a multiplex level per well on a custom array using the Sequenom MassARRAY system following the manufacturer’s instructions, which is similar to a previous study^28^. The software MassARRAY TYPER 4.0 was used to analyze the mass spectrograms and to call the SNP genotypes. Raw genotype data (cluster plots) of the SNPs were visually assessed by two investigators. All assays were performed blind to diagnosis and genotype.

### Statistical analysis in Chinese sample

Hardy–Weinberg equilibrium test was performed for all SNPs in controls using Pearson *χ*^2^-test with one degree of freedom. PLINK (v1.07) was used to analyze the statistical association between single SNPs and illness conditions, i.e., to calculate the allelic p-values, ORs and 95% confidence intervals (CIs)^29^. In the direct replication analysis of previously reported SNPs, we also applied one-tailed test in our Han Chinese sample, while in the meta-analysis of all available samples, two-tailed tests were used. Haplotype analysis was performed using the online SHEsis website (http://analysis.bio-x.cn/)^30,31^, and only haplotypes with frequencies higher than 0.001 in both cases and controls were considered. The regional association results were plotted using LocusZoom (http://locuszoom.sph.umich.edu/locuszoom/)^32^.

### Meta-analysis across different samples

After calculating the statistics of the 21 SNPs in our Han Chinese sample, we then conducted meta-analysis of data from this Chinese sample and previous published GWAS studies. Specifically, we performed meta-analysis of the 13 SNPs (rs17183814, rs2302417, rs3804640, rs11724116, rs10455979, rs10821745, rs7122539, rs12575685, rs10744560, rs4447398, rs11647445, rs112114764, and rs6130764) chosen based on the PGC2 GWAS (European ancestry)^14^ using data from our Chinese sample and the PGC2 GWAS (including both discovery and replication stages, a total of 29,764 cases and 169,118 controls, data were from Table S2 in their study^14^); for the two SNPs (rs4236274 and rs12553324) retrieved from Hou et al. GWAS^15^, the meta-analysis was performed using statistics from our Chinese sample and the original Hou et al. GWAS (including both discovery and replication stages, a total of 9784 cases and 30,471 controls, data were from Table 2 in their study^15^); the SNP rs12290811 chosen from the GWAS by Muhleisen et al.^18^ underwent meta-analysis using data obtained from our Chinese sample and their original study (including both discovery and replication stages, a total of 9747 cases and 14,278 controls, data were from Table 2 in their study^18^). We also examined five additional SNPs (rs4332037, rs78089757, rs174576, rs329674, and rs76317718) reported in the Ikeda et al. GWAS^17^. Notable, meta-analysis of rs174576, the SNP significantly linked to BPD in both Japanese and European individuals, was conducted using data from our Chinese sample, two Japanese samples, and a European study^22^ (including 20,129 cases and 21,524 controls, the sample size was different from PGC1^21^ or PGC2^14^ GWAS). Rs78089757 was not polymorphic in Europeans, and meta-analysis of the SNP was therefore conducted in solely East Asian populations, including our primary Chinese sample, three additional Chinese control sample sets, as well as two Japanese samples^17^. The meta-analysis of other three SNPs (rs4332037, rs329674, and rs76317718) was conducted using data from our Chinese sample and the data shown in Ikeda et al.^17^ (from either Table 1 or Table 2 of their study). The detailed information about the sample size and source of data for each SNP during meta-analysis is shown in Table S1. The *metafor* package in R was used to obtain pooled estimates of ORs across different samples, and to test for heterogeneity of the ORs^33^.

**Table 2:** Associations of rs174576[A-allele] with bipolar disorder in world populations

Sample	Ethnics	Case	Control	Fre_Case	Fre_Control	Two-tailed *p* value	OR	95% CIs
China	Chinese	1146	1956	0.457	0.430	0.0459	1.110	1.002–1.230
Japan 01^17^	Japanese	1545	7408	0.414	0.379	0.000181	1.160	1.073–1.255
Japan 02^17^	Japanese	1419	54,479	0.423	0.383	9.36E−06	1.190	1.102–1.285
PGC^22^	European	20,129	21,524	0.358	0.341	3.43E−06	1.074	1.042–1.108
Meta-analysis		24,239	85,367	Fixed effect model		9.33E−13	1.098	1.070–1.127
				Random effects model		2.92E−05	1.126	1.065–1.190

**Note:** Test of heterogeneity: *I*^2^ = 63.2%, *p* value = 0.0431; *OR* odds ratio, *CIs* confidence intervals

### Expression quantitative trait loci (eQTL) analyses in public datasets

We explored the impact of BPD risk SNPs on gene expression levels in human brains. For this analysis, three public gene expression datasets of genome-wide genotype data as well as RNA-seq data were used. These datasets are (1) BrainSeq^34^, (2) Genotype-Tissue Expression project (GTEx)^35^, and (3) Brain xQTL^36^.

BrainSeq (http://eqtl.brainseq.org/phase1/eqtl/) contains data of polyA + RNA-seq expression quantitative trait loci (eQTL) associations in the dorsolateral prefrontal cortex (DLPFC) tissues collected from 175 schizophrenia patients and 273 controls^34^. These individuals were all aged >13 and were mainly Caucasians and African Americans. As described in the website, the RNA-seq data was analyzed under the additive genetic effect model for the eQTL results, and was adjusted for sex, ancestry, and expression heterogeneity (principal components). We herein retrieved the results of eQTL associations from the 273 unaffected controls only. Detailed information about the BrainSeq can be found in the original publication^34^.

The GTEx (https://www.gtexportal.org/) contains information of both genetic variation and RNA-seq mRNA expression from diverse human tissues. So far, data of more than 3797 tissues from 150 postmortem donors are available for research purpose. We have thus retrieved the results of eQTL associations from the 118 frontal cortex (BA9) of healthy controls for the current study. Detailed information can be found in the original report^35^.

The Brain xQTL Serve (http://mostafavilab.stat.ubc.ca/xQTLServe/) allows assessment of the impact of genetic variation on multiple types of molecular traits derived from the human brain cortex^36^. Their samples were mainly collected using autopsies from participants of the Religious Orders Study and the Rush Memory and Aging Project. As described in this early study^36^, postmortem frozen samples of the DLPFC were obtained from these individuals, and polyA + RNA-seq gene expression (*n* = 494) analyses were performed using these samples. The effects of known (e.g., RNA integrity number, sex, age, diagnostic status, pH, PMI, and batch effects) and hidden (e.g., RNA quality and variation in cell type proportions) confounding factors were removed from the molecular phenotype data using linear regression. The authors employed Spearman’s rank correlation to estimate the association between each SNP and gene expression. This dataset provides statistical results, and the visual plots are not available in their website at the time we accessed. Detailed information can be found in the original study^36^.

### Replication of eQTL effects in Chinese brain amygdala sample

In order to replicate the observed eQTL associations of BPD risk SNPs in Chinese population, we also collected amygdala tissues of 65 nonpsychiatric controls from the Chinese Brain Bank Center. It is recognized that patients with BDP normally showed mood dysregulation as well as impaired cognitive, social, and autonomic functions^3,4,37^. Amygdala is primarily related to emotion, mood, and cognition^38^, and is therefore believed to be an important brain region in the pathogenesis of BPD. Indeed, decrease of brain amygdala volume was frequently observed in BPD patients compared to healthy controls^37,39–43^, and functional magnetic resonance imaging studies have also revealed aberrant amygdala activation in BPD patients compared with normal subjects^37,44–48^. We thus believe that this amygdala sample will provide pivotal information regarding the molecular mechanisms underlying BPD pathogenesis in Chinese population.

Total RNAs from the brain amygdala tissues were isolated by TRIzol reagent (Life technologies, USA) according to the manufacture’s protocol. The complementary DNA (cDNA) was synthesized by using a first strand cDNA synthesis kit (K1612, ThermoFisher Scientific, USA). The reverse transcription reaction mixture included 2 μg total RNA, 1.0 μL random hexamer primer, 4.0 μL 5× reaction buffer, 1.0 μL dNTP mix, 2.0 μL RiboLock RNase inhibitor, 2.0μL M-MuLV reverse transcriptase and nuclease-free water in a final volume of 20 μL. The reverse transcription reaction was incubated for 5 min at 25 °C and followed by 60 min at 42 °C. After the reverse transcription reaction, 80 μL nuclease-free water was added to make the final volume of 100 μL, for further transcription analysis. For quantitative real-time polymerase chain reaction (qRT-PCR), the reaction mixture contained 10.0 μL 2× SYBR master mix (Roche, USA), 2.0 μL primers (10 μM), 1.0 μL cDNA and 7.0 μL nuclease-free water. The qRT-PCR was performed on a 7900HT Fast Real-Time PCR System (Applied Biosystems, USA) following the program that firstly 95 °C for 10 min, followed by 40 repeated cycles of 95 °C for 15 s and 60 °C for 30 s. The *FADS1* expression was determined by relative quantitative analysis and *RPS13* was employed as an internal control gene. The relative gene expression was presented as the means of –ΔCt for a statistical test against genotypic groups^49^, and the *p* values were calculated using one-way ANOVA. The sequences of primers used for amplification of *RPS13* were 5′-CCCCACTTGGTTGAAGTTGA-3′ (forward) and 5′-CTTGTGCAACACCATGTGAA-3′ (reverse), and sequences of primers for *FADS1* were 5′-TATATGCCGTACAACCACCAGC-3′ (forward) and 5′-GAAGCGGACGTAGAAGGTAATCA-3′ (reverse).

### Cross-disorder analysis using statistical data from CONVERGE GWAS

Previous studies have suggested substantial genetic overlap between BPD and major depressive disorder (MDD)^50–53^. To investigate if the aforementioned BPD risk SNPs were also associated with risk of MDD in Han Chinese population, we utilized data of Han Chinese individuals from the previously published CONVERGE MDD GWAS^10^. In brief, the CONVERGE GWAS consisted of 5303 MDD cases and 5337 mentally healthy controls, in which the MDD cases were diagnosed according to the DSM-IV criteria^10^. As described earlier, controls were recruited either from local communities or from the patients who underwent minor surgical procedures at the general hospitals. Detailed information about the sample composition, quality control, and statistical methods can be found in the original study^10^.

## Results

Of the 3102 individuals genotyped, 1794 were male and 1308 were female. The final genotyping rate was 0.9855. The allele and genotype frequencies of the 21 SNPs are shown in Table 1 and Table S2. Sixteen of the 21 (76.2%) SNPs exhibited the same direction of allelic effects regarding their roles in BPD susceptibility in our Han Chinese sample compared with previous GWAS. Six SNPs had nominal significant associations with the illness (*p* < 0.05, Table 1) at one-tailed test in our Chinese samples, and two additional SNPs showed marginal significance (*p* < 0.10, Table 1). Meta-analysis combining our Chinese samples with data of previous studies yielded genome-wide significant associations for 15 these tested SNPs (two-tailed *p* < 5.00E−08, Table S1).

### *FADS1* is a risk gene for bipolar disorder

Our study, rather than providing solely the above replication results of previously reported SNPs, has also highlighted several SNPs for further analyses. First, rs174576 in *FADS1/2* region, the SNP showing genome-wide significant association in the meta-analysis of Japanese and European PGC1 GWAS samples (two-tailed *p* = 1.34E−10, OR = 1.130)^17^, is also significantly associated with BPD in our Han Chinese sample (one-tailed *p* = 0.0229, OR = 1.110, Table 1). When our Han Chinese samples were combined with data from previous GWAS samples of Japanese^17^ (2964 cases and 61,887 controls) and Europeans^22^ (20,129 cases and 21,524 controls) for further meta-analysis, a stronger genome-wide association between rs174576 and BPD was seen under fixed effect model (two-tailed *p* = 9.33E−13, OR = 1.098, Table 2).

The allele frequencies of rs174576 in European and East Asian populations are similar (Figure S1), and in both populations there are multiple SNPs in high linkage disequilibrium (LD) with rs174576 (*r*^2^ > 0.8, Figure S2). Although it is difficult to determine the potential causative variant for this locus, functional prediction in GWAVA dataset (http://www.sanger.ac.uk/sanger/StatGen_Gwava)^54^ and HaploReg (http://archive.broadinstitute.org/mammals/haploreg/haploreg.php)^55^ showed several SNPs deserving further investigation (Table S3 and Figure S3).

**Fig. 1:**
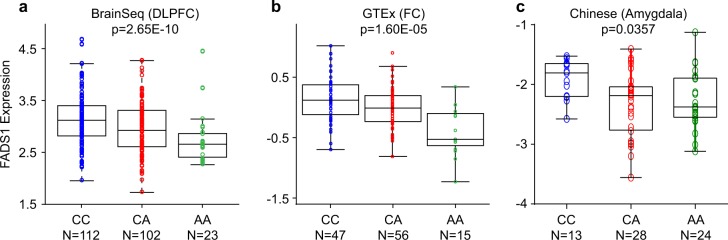
Association of rs174576 with *FADS1* mRNA expression in BrainSeq (**a**), GTEx (**b**), and our own Chinese sample (**c**). DLPFC dorsolateral prefrontal cortex, FC frontal cortex

To gain more insights into this issue, we conducted eQTL analysis in brain tissues as it is proven to be effective in discovering the underlying molecular mechanism of risk association^56–58^. This approach has little dependence on the causative variant, and LD SNPs of the causative variant could also show eQTL associations with the relevant gene expression^24,25^. Briefly, eQTL analysis of rs174576 with nearby gene expression in brain samples was performed using BrainSeq as the discovery sample^34^. In this dataset, we observed that rs174576 was significantly associated with the gene expression of *FADS1* (two-tailed *p* = 2.65E−10, Fig. 1), with the BPD risk allele [A] predicting lower levels of *FADS1* mRNA. We then examined the eQTL association between rs174576 and *FADS1* using the GTEx and Brain xQTL datasets^35,36^, and the above finding was reproduced (two-tailed *p* = 1.60E−05 in GTEx and two-tailed *p* = 6.77E−14 in Brain xQTL, Fig. 1 and Table S4, respectively), with the eQTL associations in the three datasets all in the same directions. We then conducted eQTL analysis between rs174576 and *FADS1* expression in our Han Chinese amygdala samples (*n* = 65) to validate if this eQTL association exists across different ethnic populations, and more intriguingly, we found that rs174576 was also significantly associated with *FADS1* expression in Han Chinese (two-tailed *p* = 0.0357, Fig. 1). Together, these data strongly support the important role of rs174576 and *FADS1* in BPD pathogenesis, and the causal factors among rs174576 and its linked SNPs remains to be determined. We also tested the association between rs174576 and the expression of *FADS2* in the above mRNA datasets, but did not see significant associations (Tables S4 and S5). In the Brain xQTL dataset, rs174576 was also significantly associated with *TMEM258* expression (Table S4), but this association was not replicated in GTEx or BrainSeq database (Table S5), and was therefore not followed up in our Chinese amygdala sample.

### *MAD1L1* SNPs and haplotypes are associated with bipolar disorder

Besides rs174576, SNPs (rs4236274 and rs4332037) in *MAD1L1* also worth further investigations. Rs4236274 and rs4332037 are two SNPs in close proximity (54.4 kb between each other, their positions are shown in Figure S4) in the intron regions of *MAD1L1*. The two SNPs are in weak LD in both European and East Asian populations from 1000 Genomes Project^59^ (both *r*^2^ = 0.085), which was further seen in our own Han Chinese samples (*r*^2^ = 0.123). More importantly, the two (partially) independent SNPs have been reported to be genome-wide significantly associated with BPD in different GWAS studies^15,17^. In Hou et al. GWAS comprising of all European individuals^15^, rs4236274 showed genome-wide significant associations (two-tailed *p* = 8.49E−12, OR = 1.149, Table 1 and Figure S4), while rs4332037 only displayed nominal associations (two-tailed *p* = 0.00392, Figure S4). In Ikeda et al.^17^ GWAS including Japanese and European populations, rs4332037 was genome-wide significantly associated with BPD (two-tailed *p* = 1.91E−09, OR = 1.170, Table 1). In the current Han Chinese sample, both SNPs exhibited nominal significant associations with BPD (rs4236274, one-tailed *p* = 0.00731, OR = 1.140; rs4332037, one-tailed *p* = 0.0104, OR = 1.218; Table 1) in the same allelic directions and similar effect sizes compared with the previous GWAS studies^15,17^.

Given that rs4236274 and rs4332037 are in low LD, we also conducted the haplotype analysis of these two SNPs in our Han Chinese sample, and found that a common protective haplotype (G–C) showed significantly lower frequency in BPD cases (0.518) compared with controls (0.553) (haplotype *p* = 0.00845, OR = 0.868, Fisher’s exact test, Table 3). Global test also confirmed the haplotype analysis results (Global *p* = 0.00786, Fisher’s exact test). These results suggest that there are likely (at least partially) independent risk association signals in the *MAD1L1* region, and there might be other causative variants remains to be identified. Overall, our single SNP and haplotype analyses support that *MAD1L1* is a risk locus for BPD in Chinese population. However, neither of these *MAD1L1* SNPs are associated with any gene expression in brains in the published eQTL datasets (data not shown)^34–36^, further studies elucidating their functional impacts are thus needed.

**Table 3:** Haplotype analyses of *MAD1L1* rs4236274-rs4332037 with bipolar disorder in Chinese populations

Haplotype	Frequency		*χ* ^2^	Fisher’s *p* value	Pearson’s *p* value	OR	95% CIs
	Case	Control					
A–C	0.371	0.350	2.597	0.107	0.107	1.094	0.981–1.220
A–T	0.107	0.095	2.033	0.154	0.154	1.134	0.954–1.349
G–C	0.518	0.553	6.940	**0.00846**	**0.00845**	0.868	0.782–0.964
G–T	0.004	0.001	5.202	**0.0226**	**0.0226**	3.261	1.112–9.557

Global result: Global *χ*^2^ is 11.879 while d*f* = 3 (frequency < 0.001 in both control and case has been dropped)

Fisher’s *p* value is 0.00786; Pearson’s *p* value is 0.00781. *OR* odds ratio, *CIs* confidence intervals

### A rare variant rs78089757 is genome-wide significantly associated with bipolar disorder in East Asians

In addition, we have also replicated the nominal or marginal significant associations at several other loci, such as rs2302417 in *ITIH3* and rs17183814 in *SCN2A* (one-tailed *p* value < 0.10, Table 1). Among these, there is a SNP rs78089757 located in 10q26.13 (intergenic region), which has been previously reported in a Japanese GWAS^17^, did not achieve genome-wide significance (two-tailed *p* = 3.99E−07, OR = 1.410, Table 1) probably due to the limited sample size. According to previous studies^17^, this SNP is rare in Japanese population (minor allele frequency < 0.05), and is almost monomorphic in European populations (not included in the current GWAS of BPD in European populations probably due to its low frequency). We thus checked the data from 1000 Genomes Project^59^ and confirmed that rs78089757 is also a rare SNP in Chinese population. We then performed further tests to understand its role in the genetic risk of BPD in Chinese, and found that rs78089757 was also nominally associated with BPD in 1146 cases and 1956 controls (one-tailed *p* = 0.0440, OR = 1.358, Table 1), with similar effect size as that in the Japanese GWAS^17^. To maximize the statistical power of our analyses, we then collected all available genotypic data through combining Han Chinese control subjects from 1000 Genomes Project^59^ (*n* = 208 individuals), CONVERGE Consortium^10^ (*n* = 5222 subjects), and an additional control cohort (*n* = 1908 individuals) with our own Han Chinese sample, and the association became stronger in the combined analysis (one-tailed *p* = 0.00176, OR = 1.526 in a total of 1146 cases and 9294 controls, Table 4). A meta-analysis combining Han Chinese data with Japanese GWAS^17^ showed that rs78089757 was genome-wide significantly associated with BPD (two-tailed *p* = 5.22E−09, OR = 1.429, Table 4), indicating that it is likely a risk SNP in East Asian populations. While rs78089757 could be an East Asian specific risk locus, but whether it is involved in BPD susceptibility in European populations remains unclear. Current European GWAS studies have not covered this SNP, and its surrounding genetic variations were not associated with BPD in European populations (the statistical data was from Hou et al. GWAS^15^, a total of 3095 SNPs in this region were included, and the regional-wide corrected *p* value should be 0.05/3095 = 1.62E−05, Figure S5).

**Table 4:** Association of rs78089757[A-allele] with bipolar disorder in East Asian populations

Sample	Ancestry	Case	Control	Fre_Case	Fre_Control	Two-tailed *p* value	OR	95% CIs
China	Chinese	1146	1956	0.0255	0.0191	0.0881	1.358	0.955–1.931
1KGenome^59^	Chinese		208		0.00962			
CONVERGE^10^	Chinese		5222		0.0160			
China	Chinese		1908		0.0183			
Combined Chinese		1146	9294	0.0255	0.0169	0.00352	1.526	1.149–2.028
Japan 01^17^	Japanese	1545	7408	0.041	0.032	0.00627	1.330	1.087–1.628
Japan 02^17^	Japanese	1419	54,479	0.050	0.035	1.44E−05	1.470	1.235–1.750
Meta-analysis		4110	71,181	/	/	5.22E−09	1.429	1.268–1.610

Note: Test of heterogeneity: *I*^2^ = 0, *p* value = 0.673. *OR* odds ratio, *CIs* confidence intervals; 1KGenome, 1000 Genomes Project. The genotype data of rs78089757 in the 1000 Genomes Project includes 103 CHB (Han Chinese in Beijing, China) and 105 CHS (Southern Han Chinese, China) subjects. The additional Chinese control individuals (*n* = 1908) have not been published before

### ***SCN2A*and*SHANK2*are associated with major depressive disorder in Han Chinese**

Given the substantial genetic overlap between BPD and MDD^50–53^, we also examined the associations of the 21 BPD risk SNPs with MDD in a published Chinese GWAS conducted by CONVERGE Consortium (5303 cases and 5337 controls)^10^. Among the 21 tested SNPs, 18 European BPD risk alleles were also highlighted in Chinese MDD cases compared with controls. Importantly, two SNPs (rs17183814 and rs12575685) have even achieved nominal significance (Table S6). Rs17183814 locates in the gene *SCN2A*. This SNP was previously reported to confer genome-wide significant risk of BPD in Europeans (two-tailed *p* = 2.02E−09, OR = 1.142, Table 1)^14^, and is also associated with BPD in our Han Chinese sample with the similar effect size (one-tailed *p* = 0.0311, OR = 1.153, Table 1). We reveal that the BPD risk allele [G] of the SNP also increases risk of MDD in a similar effect size (two-tailed *p* = 0.00875, OR = 1.116, Table S6). The second SNP rs12575685 locates in the gene *SHANK2*, and is also significantly associated with MDD in Chinese (two-tailed *p* = 0.00558, OR = 1.095, Table S6). However, it did not show evidence of association with BPD in our Chinese sample (one-tailed *p* = 0.143, OR = 1.066, Table 1). The considerable OR for this SNP suggests that this negative result is probably due to the limited sample size.

## Discussions

In the current studies, we have performed replications of previous BPD GWAS findings in an independent case and control sample set of Chinese origin. By combining our data with the previous BPD GWAS datasets, we have further confirmed that several genes (such as *FADS1*, *MAD1L1*, *SCN2A*, and *ITIH3*) are likely involved in the genetic risk of BPD. We also observed a genome-wide significant locus at 10q26.13 in East Asian populations.

This study has provided essential information as we have used a newly collected BPD sample from Mainland China, which has not been explored before the year of 2018. While we have tested whether the risk associations observed in other populations could be replicated in Chinese, further expanding analyses are also warranted to uncover Chinese specific genetic risk factors for BPD. Additionally, we have also utilized the brain amygdala samples from Chinese population. Although the sample size is relatively moderate compared to European brain eQTL studies^34,36,56^, it is still one of the largest brain samples ever reported in Chinese population. These data are therefore valuable in helping elucidate the molecular mechanisms underlying genetic risk factors of BPD and other psychiatric illnesses.

We have reported the intriguing finding of the association between rs174576 and *FADS1* expression. Indeed, this genomic region has been recently highlighted in several BPD GWAS^14,17^. However, this genomic region contains numerous high-LD SNPs and spans many genes (Figure S2), making it difficult to pinpoint the causative gene(s). Our analyses, together with previous studies, suggest that *FADS1* is likely a BPD-related gene, and decreased expression of *FADS1* might be a potential risk factor. However, this gene was not highlighted in recent studies of genome-wide RNA-seq analyses in the brains of BPD patients^60–63^, and in a candidate gene study, mRNA expression of *FADS1* was slightly higher in patients with BPD compared with nonpsychiatric controls^64^. Therefore, the mechanism by which *FADS1* involved in BPD pathogenesis is rather more complicated and remains to be elucidated. The *FADS1* gene encodes a member of the fatty acid desaturase (FADS) protein family. As described previously, *FADS1* desaturates omega-3 and omega-6 polyunsaturated fatty acids at the delta-5 position and thereby catalyzes the final step of eicosapentaenoic acid (EPA) and arachidonic acid formation^65^. Interestingly, the SNP rs174576 has been reported to correlate with whole-body fat oxidation^66^, desaturase indices, and the impact of hormonal contraceptives on plasma docosahexaenoic acid concentrations^67,68^. While long-chain (>20 C-atoms) polyunsaturated fatty acids (LC PUFAs) are known to be important for the functional integrity of brain development, cognitive abilities, and mood^69,70^, the association between rs174576 and psychiatric disorders gains further support. These lines of evidence are in line with a previous study^17^, in which the authors speculated that alterations in lipid signaling pathways and metabolism might be involved in the pathophysiology of BPD. In fact, epidemiological studies found that increased incidences of hyperlipidemia and metabolic syndrome appeared in BPD patients compared with general populations^71–73^. In addition, a previous study found that rs174576 was associated with white matter abnormality in 83 preterm infants (quantified in vivo using diffusion tensor imaging), providing possible evidence that susceptibilities to BPD later in life may be linked to the in uterus exposure to environmental stress^74^.

Another gene implicated in the current study, *MAD1L1*, encodes the mitotic spindle assembly checkpoint protein MAD1 that facilitates appropriate onset of anaphase after proper alignment of all chromosomes at the metaphase plate^75^. MAD1L1 functions as a homodimer. By interacting with MAD2L1, HDAC1, and histone deacetylase 2^76,77^, this protein likely plays a role in cell cycle control and tumor suppression^78^. The *MAD1L1* gene is also known as human accelerated region 3 (HAR3; as mentioned in previous studies^79,80^, HARs are a set of 49 human genomic segments that are conserved throughout vertebrate evolution but are sharply different in human beings), and thus may have played a key role in human evolution^79,80^. Intriguingly, *MAD1L1* has also been implicated in the genetic susceptibility to schizophrenia^9,11,81,82^, another psychiatric disease sharing substantial risk factors with BPD. It was also found that *MAD1L1* was linked to the reward systems functioning in healthy adults (an intermediate phenotype for BPD and schizophrenia)^83^, adding further evidence for its involvement in psychiatric abnormalities. Although the exact function of *MAD1L1* in brain development and even these illnesses remains unclear, the above evidence strongly suggests that the acceleration of *MAD1L1* region in humans could play pivotal roles in the arising of these human-specific or human-dominant diseases (i.e., BPD and schizophrenia). Further studies dissecting the underlying mechanisms are therefore necessary.

We have also replicated the nominal or marginal associations between variants at *ITIH3, SCN2A*, and *CD47* and the risk of BPD in our Han Chinese sample. *ITIH3* is located at chromosome 3p21, the genomic region repeatedly showing significant associations with BPD^14,15,17,20,84,85^ and other mental illnesses^9,11,86,87^. This chromosome 3p21 region contains multiple high-LD variants in a tight cluster of genes, and thus a causative gene is yet to be determined. *SCN2A* encodes sodium voltage-gated channel alpha subunit 2 (Na_v_1.2). Although this gene has not been highlighted in earlier BPD studies, de novo mutations in *SCN2A* were found associated with autisms^88–91^, and common variations within or near this gene were also associated with cognitive performance in schizophrenia patients^92^. Besides, the gene *CACNA1C* also deserved further attention. In the current study, the *CACNA1C* SNP rs10744560 did not achieve nominal significance in our Chinese sample (*p* = 0.143), however, its OR in our sample is consistent with that in European GWAS^14^ (1.130 in Chinese versus 1.076 in Europeans), suggesting that the size of our sample is likely too small to reveal the true significant association between this SNP and BPD. The genome-wide significant association in the meta-analysis of our Chinese sample and previous studies also support this conclusion. *CACNA1C* is a promising BPD candidate gene that encodes the Cav1.2 subunit of voltage-gated calcium channels^93,94^; it is one of the best replicated genetic susceptibility genes for BPD^14,18,19,21^ and other psychiatric disorders^9,11,52,95–97^ across distinct populations, and the risk gene also affects the disease relevant phenotypes in humans^98–100^ and in animal models^101–103^ as implicated by multiple studies. Our results thus strongly supports the involvement of these genes in the genetic risk of BPD, but further replication studies are also necessary.

There are, however, several limitations in the present study and we are cautious about the interpretation of these results. First, we replicated the eQTL association between rs174576 and *FADS1* expression in our Han Chinese amygdala sample using qRT-PCR techniques, while in the three European eQTL datasets, they all utilized RNA-seq methods. It is therefore interesting to examine the variation of gene expression quantification results using different methods, and future replication of the eQTL associations using RNA-seq in our Chinese amygdala sample is needed. Second, although we have excluded individuals with histories of major neuropsychiatric or neurological disorders in the control samples, there are the possibilities that some control individuals might also have other types of mental illness, such as cyclothymic disorder (this illness was not individually included in our BPD patients sample)^104^, although the prevalence of those illnesses in general populations is relatively low and might not significantly alter the association results.

In summary, we have provided additional support for prior association findings in *FADS1*, *MAD1L1*, *SCN2A*, and *ITIH3*, and have confirmed a risk locus at 10q26.13 that is associated with BPD. While we have presented the genetic association study of BPD using the Han Chinese samples recruited in Mainland China, and have explored relevant mechanisms using the cohort of brain amygdala samples of Chinese origin, the samples sizes are yet to be enlarged for better resolution and statistical power.

## Supplementary information

Supplementary Figures
Supplementary Tables
