# Supplementary material for: Replicated associations of FADS1, MAD1L1, and a rare variant at 10q26.13 with bipolar disorder in Chinese population
Source: Transl Psychiatry. 2018 Dec 7;8:270. doi: 10.1038/s41398-018-0337-x (PMC6286364; doi:10.1038/s41398-018-0337-x)

Figure S1. Global distributions of rs174576 in 53 world populations

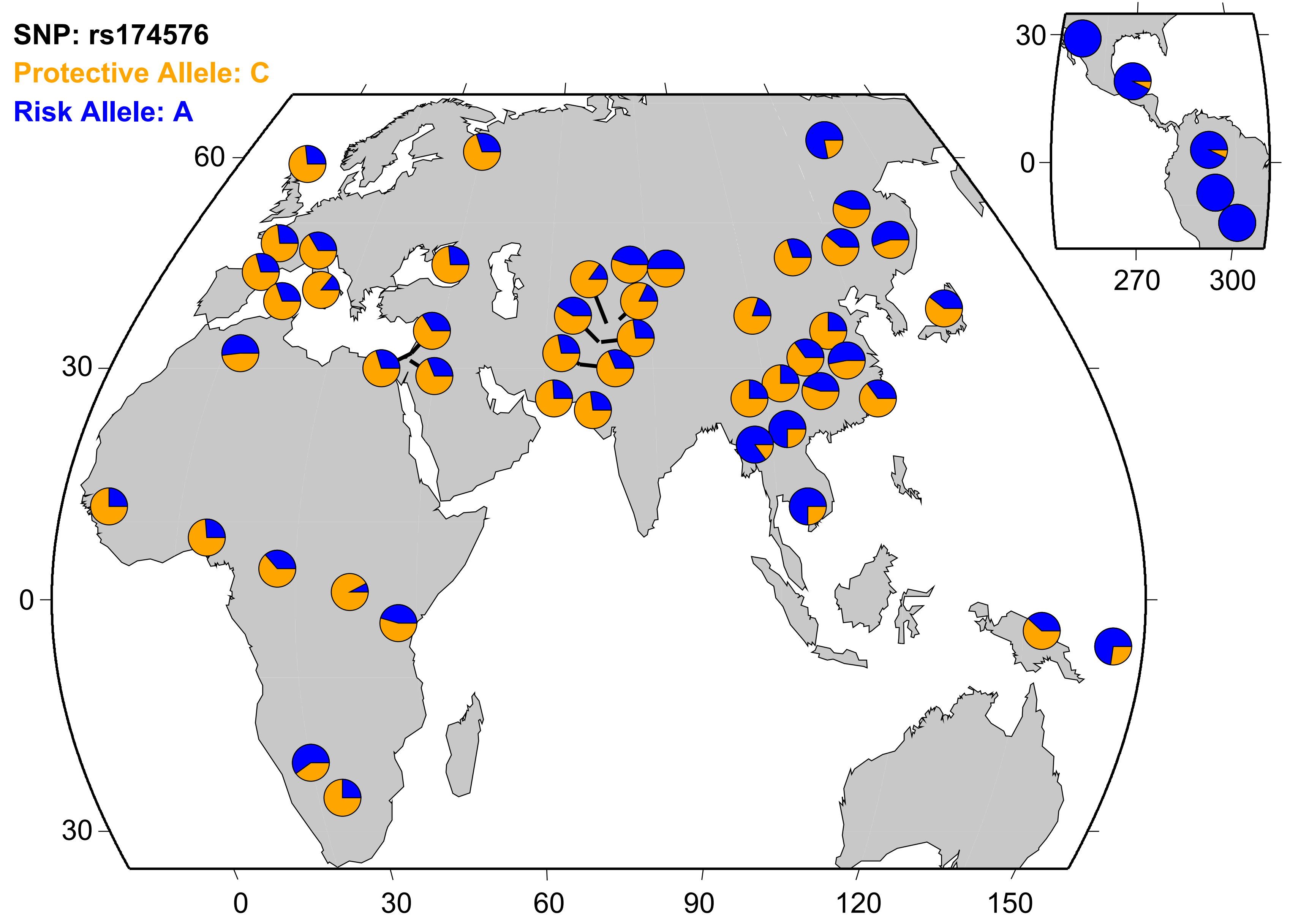

Figure S2. Plot of chromosome region showing a genomic area of LD with rs174576 in Europeans and East Asians

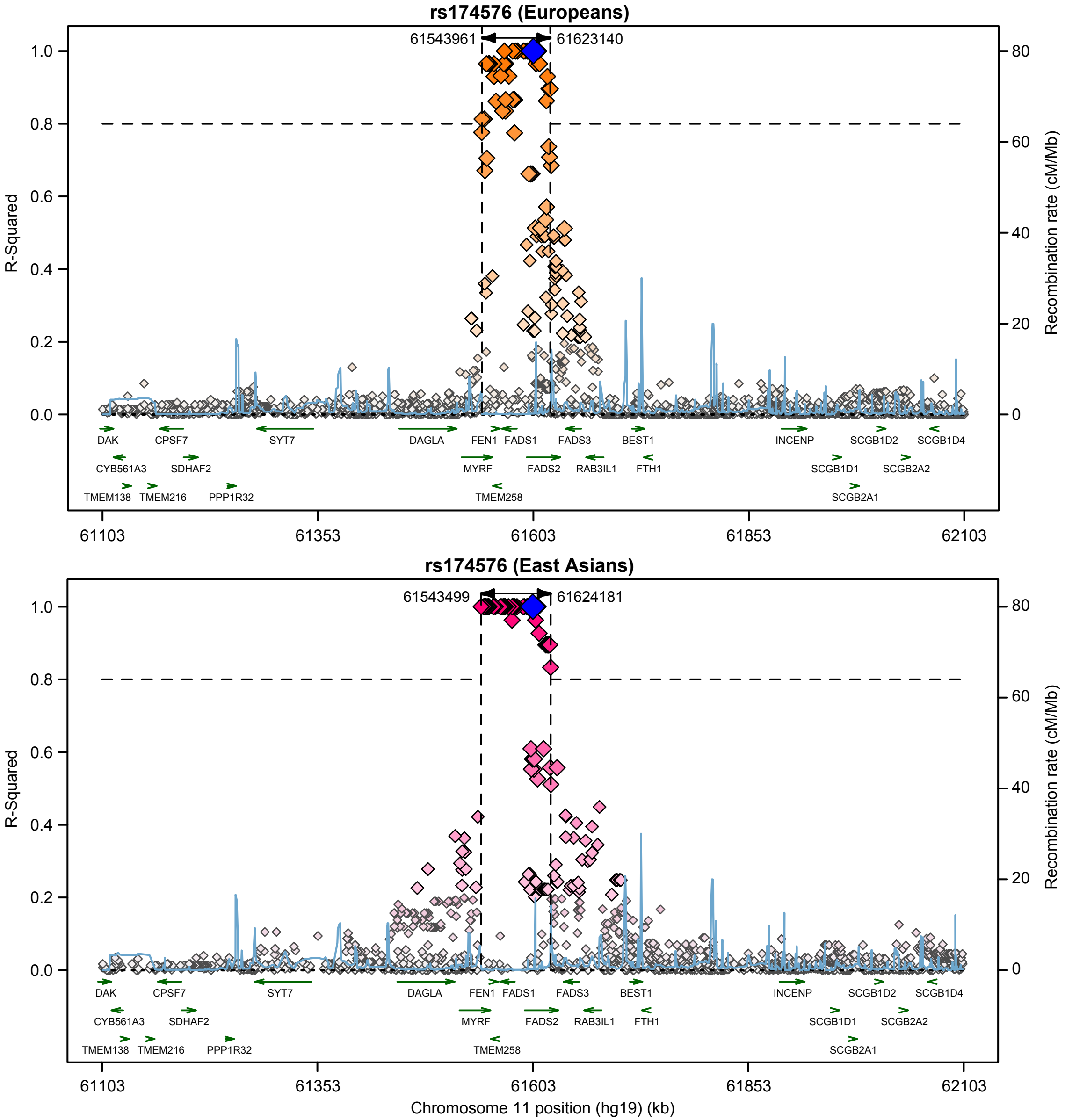

Figure S3. Functional prediction of the SNPs in high LD (r2>0.8) with rs174576 using HaploReg

Query SNP: rs174576 and variants with r^2 >= 0.8

chr	pos (hg38)	LD (r^2)	LD (D')	variant	Ref	Alt	AFR freq	AMR freq	ASN freq	EUR freq	SiPhy cons	Promoter histone marks	Enhancer histone marks	DNAse	Proteins bound	Motifs changed	NHGRI/EBI GWAS hits	GRASP QTL hits	Selected eQTL hits	GENCODE genes	dbSNP func annot
11	61781553	0.85	0.94	rs174533	G	A	0.04	0.55	0.41	0.36		SKIN	7 tissues	LNG		PU.1		16 hits	42 hits	C11orf9	intronic
11	61783884	0.85	0.93	rs174535	T	C	0.14	0.56	0.41	0.36			7 tissues	ESC,KID,BRN	CTCF	Mrg	6 hits	115 hits	45 hits	C11orf9	synonymous
11	61784455	0.86	0.94	rs174536	A	C	0.04	0.55	0.41	0.36			8 tissues	LIV		HEN1,ZBTB7A	2 hits	114 hits	44 hits	C11orf9	intronic
11	61785208	0.86	0.94	rs174537	G	T	0.04	0.55	0.41	0.36			ESDR, IPSC, SKIN			HNF4	2 hits	705 hits	44 hits	C11orf9	intronic
11	61790331	0.88	0.95	rs102275	T	C	0.63	0.63	0.41	0.37							11 hits	156 hits	41 hits	C11orf10	intronic
11	61790354	0.86	0.95	rs102274	T	C	0.04	0.55	0.41	0.36						EWSR1-FLI1		16 hits	41 hits	C11orf10	intronic
11	61801834	0.87	0.95	rs174545	C	G	0.04	0.54	0.41	0.36			ESDR, LNG, BLD	IPSC		4 altered motifs	2 hits	110 hits	44 hits	FADS1	3'-UTR
11	61802358	0.87	0.95	rs174546	C	T	0.04	0.54	0.41	0.36			ESDR, LNG, BLD	BLD		BDP1,RXRA	11 hits	159 hits	44 hits	FADS1	3'-UTR
11	61803311	0.87	0.95	rs174547	T	C	0.04	0.54	0.41	0.36			SKIN, BLD			AIRE,Egr-1	13 hits	399 hits	44 hits	FADS1	intronic
11	61804006	0.87	0.95	rs174550	T	C	0.04	0.54	0.41	0.36			4 tissues		POL2,POL24H8	ERalpha-a	10 hits	116 hits	44 hits	FADS1	intronic
11	61806212	0.8	0.94	rs174551	T	C	0.04	0.48	0.38	0.34			BLD			8 altered motifs			41 hits	FADS1	intronic
11	61807686	0.87	0.95	rs174553	A	G,T	0.04	0.54	0.41	0.36			BLD	MUS,BLD				16 hits	41 hits	FADS1	intronic
11	61811991	0.87	0.95	rs174554	A	G	0.04	0.54	0.41	0.36			GI, BLD	4 tissues		4 altered motifs		16 hits	41 hits	FADS1	intronic
11	61817672	0.87	0.95	rs174562	A	G	0.04	0.54	0.41	0.36		19 tissues	14 tissues	11 tissues		Znf143		16 hits	41 hits	FADS1	
11	61820833	0.87	0.95	rs174564	A	G	0.08	0.56	0.41	0.36			12 tissues			4 altered motifs			40 hits	FADS1	
11	61824890	0.91	0.96	rs174566	A	G	0.20	0.57	0.41	0.36		SKIN	BLD					16 hits	40 hits	FADS1	
11	61825533	0.9	0.96	rs174567	A	G	0.21	0.57	0.41	0.36						HNF4,RXRA,VDR			38 hits	FADS1	
11	61826344	0.88	0.96	rs174568	C	T	0.04	0.54	0.41	0.35			9 tissues	MUS,LIV				16 hits	43 hits	FADS1	
11	61827448	0.88	0.95	rs3834458	CT	C	0.04	0.54	0.41	0.36		21 tissues	7 tissues	32 tissues	4 bound proteins	4 altered motifs			42 hits	FADS1	
11	61828850	0.88	0.96	rs5792235	CA	C	0.10	0.55	0.41	0.36		22 tissues	BLD, GI	25 tissues	5 bound proteins	9 altered motifs			41 hits	FADS1	intronic
11	61829161	0.91	0.96	rs99780	C	T	0.35	0.57	0.41	0.37		22 tissues	4 tissues	34 tissues	POL2	4 altered motifs		16 hits	39 hits	FADS1	intronic
11	61830500	0.9	0.96	rs1535	A	G	0.11	0.55	0.41	0.36		18 tissues	15 tissues	7 tissues	ERALPHA_A	AIRE,ERalpha-a,Nrf1	5 hits	152 hits	45 hits	FADS2	intronic
11	61832870	0.93	-0.97	rs174574	A	C	0.44	0.39	0.59	0.63		BLD	15 tissues	7 tissues	POL2	4 altered motifs	2 hits	20 hits	43 hits	FADS2	intronic
11	61836038	1	1	rs174576	C	A	0.27	0.58	0.41	0.37		BLD	7 tissues	IPSC,BLD		4 altered motifs	1 hit	130 hits	40 hits	FADS2	intronic
11	61837342	0.98	1	rs174577	C	A	0.34	0.59	0.41	0.37			5 tissues	4 tissues		Zic	3 hits	115 hits	41 hits	FADS2	intronic
11	61838027	0.98	1	rs174578	T	A	0.34	0.59	0.41	0.37		SPLN	IPSC, BLD		STAT1,STAT2	4 altered motifs	3 hits	17 hits	41 hits	FADS2	intronic
11	61839170	0.98	1	rs174580	A	G	0.34	0.59	0.41	0.37				BLD		4 altered motifs		16 hits	37 hits	FADS2	intronic
11	61839211	0.98	1	rs174581	G	A	0.34	0.59	0.41	0.37				BLD		VDR		16 hits	37 hits	FADS2	intronic
11	61842278	0.94	0.98	rs174583	C	T	0.28	0.59	0.42	0.37		BLD	4 tissues	4 tissues	POL2	Nkx2	3 hits	118 hits	39 hits	FADS2	intronic
11	61843278	0.9	0.95	rs174584	G	A	0.37	0.59	0.42	0.37			BLD			7 altered motifs		16 hits	36 hits	FADS2	intronic
11	61851136	0.85	0.97	rs174592	A	G	0.58	0.61	0.42	0.39		BRN	8 tissues	6 tissues		6 altered motifs		16 hits	35 hits	FADS2	intronic
11	61852357	0.82	-0.96	rs174594	C	A	0.08	0.35	0.58	0.61			ADRL, HRT	SKIN		Nanog		16 hits	34 hits	FADS2	intronic
11	61854084	0.81	0.94	rs174599	G	C	0.63	0.62	0.42	0.39			11 tissues	5 tissues		RXRA		16 hits	32 hits	FADS2	intronic
11	61855668	0.82	0.96	rs174601	C	T	0.63	0.62	0.42	0.40			6 tissues				4 hits	19 hits	37 hits	FADS2	intronic

Figure S4. Locations of rs4236274 and rs4332037 in *MAD1L1*. The figure was plotted *via* LocusZoom using statistical data from Hou et al. BPD GWAS.

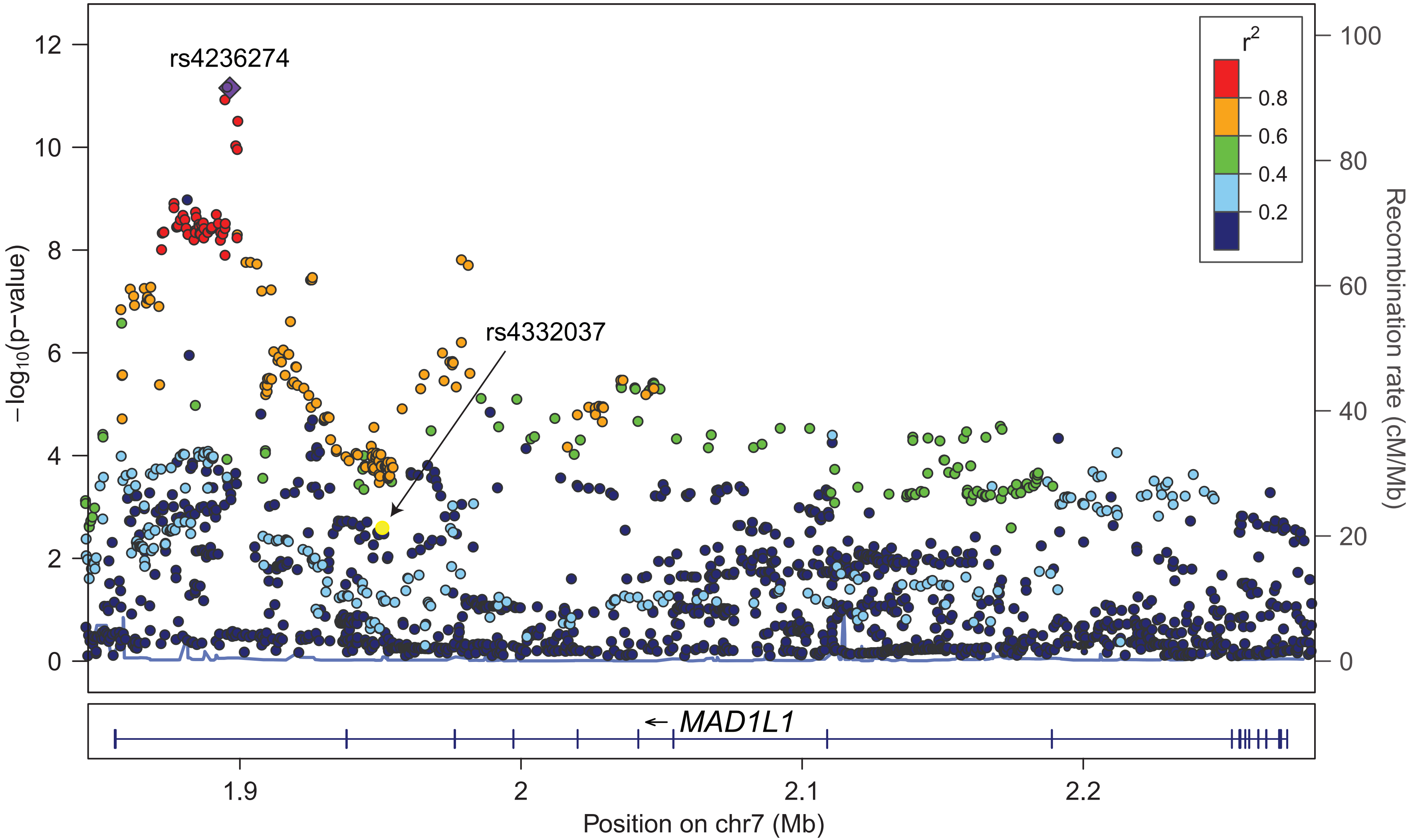

Figure S5. Associations of genetic variations surrounding rs78089757 with BPD in European populations. The figure was plotted *via* LocusZoom using statistical data from Hou et al. BPD GWAS.

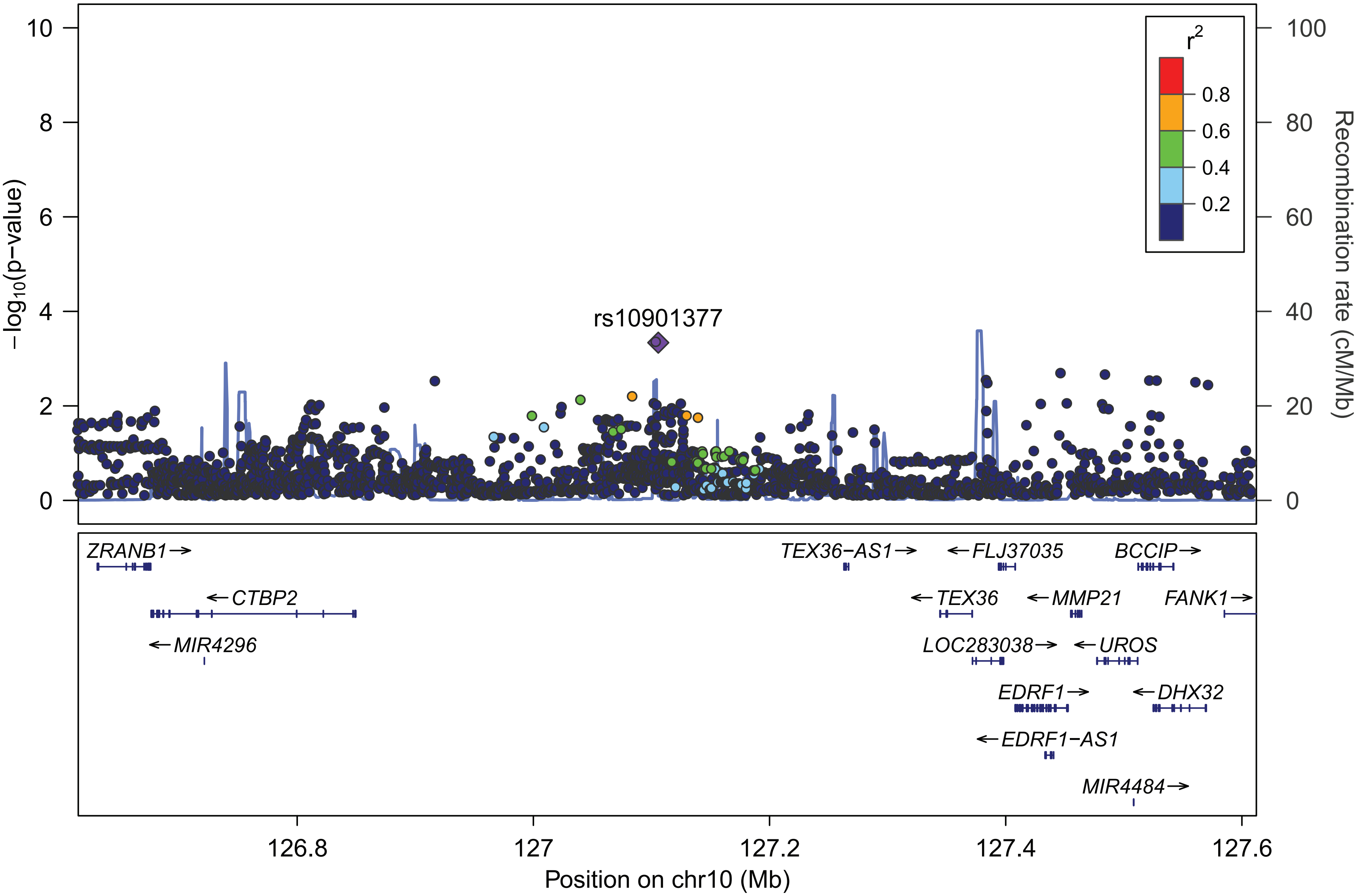

Supplement: Supplementary file 1 — Supplementary Figures [file 41398_2018_337_MOESM1_ESM.pdf]
