# Supplementary material for: Replicated associations of FADS1, MAD1L1, and a rare variant at 10q26.13 with bipolar disorder in Chinese population
Source: Transl Psychiatry. 2018 Dec 7;8:270. doi: 10.1038/s41398-018-0337-x (PMC6286364; doi:10.1038/s41398-018-0337-x)
Supplement: Supplementary file 2 — Supplementary Tables [file 41398_2018_337_MOESM2_ESM.pdf]

Table S1. Meta-analysis of the 21 SNPs using our Han Chinese samples with previous GWAS.

CHR	SNP	Allele	Our Han Chinese			Previous studies						Meta-analysis			
			OR	SE	P-value	Case	Control	OR	SE	P-value	Ref.	P-value	OR	Q	I^2
2	rs17183814	G/A	1.153	0.077	0.0623	29,764	169,118	1.142	0.022	2.02E-09	14	3.47E-10	1.143	0.902	0
3	rs2302417	T/A	1.083	0.054	0.144	29,764	169,118	1.076	0.011	6.59E-11	14	1.89E-11	1.076	0.909	0
3	rs3804640	A/G	1.147	0.077	0.0738	29,764	169,118	1.065	0.011	1.99E-08	14	6.42E-09	1.067	0.339	0
4	rs11724116	C/T	1.045	0.077	0.571	29,764	169,118	1.089	0.015	2.37E-08	14	2.18E-08	1.087	0.602	0
6	rs10455979	G/C	0.944	0.060	0.336	29,764	169,118	1.064	0.011	4.31E-08	14	0.777	1.017	0.048	74.40
7	rs4236274	A/G	1.140	0.054	0.0146	9,784	30,471	1.149	0.020	8.49E-12	15	4.09E-13	1.148	0.886	0
7	rs4332037	T/C	1.218	0.086	0.0209	10,445	71,137	1.170	0.026	1.91E-09	17	1.38E-10	1.174	0.653	0
9	rs12553324	G/C	0.946	0.053	0.297	9,784	30,471	1.120	0.019	5.87E-09	15	0.668	1.037	0.003	88.79
10	rs10821745	G/T	1.013	0.063	0.835	29,764	169,118	1.145	0.023	6.76E-09	14	0.140	1.092	0.067	70.20
10	rs78089757	A/G	1.358	0.180	0.0881	The result of meta-analysis of this SNP is shown in **Table 3**									
11	rs174576	A/C	1.110	0.052	0.0459	The result of meta-analysis of this SNP is shown in **Table 2**									
11	rs7122539	G/A	1.086	0.052	0.113	29,764	169,118	1.067	0.012	3.77E-08	14	1.29E-08	1.068	0.739	0
11	rs12575685	A/G	1.066	0.060	0.285	29,764	169,118	1.073	0.012	7.71E-09	14	4.85E-09	1.072	0.918	0
11	rs12290811	A/T	1.093	0.112	0.428	9,747	14,278	1.190	0.027	7.81E-11	18	7.48E-11	1.185	0.460	0
11	rs329674	A/G	0.973	0.066	0.678	2,964	61,887	1.220	0.037	9.59E-08	17	0.414	1.097	0.003	88.79
12	rs10744560	T/C	1.130	0.115	0.287	29,764	169,118	1.076	0.012	3.62E-10	14	2.41E-10	1.077	0.671	0
15	rs4447398	A/C	0.977	0.060	0.701	29,764	169,118	1.099	0.016	9.37E-09	14	0.386	1.051	0.058	72.29
16	rs11647445	G/T	1.003	0.099	0.978	29,764	169,118	1.079	0.012	1.08E-10	14	1.36E-10	1.077	0.466	0
17	rs76317718	G/T	0.989	0.058	0.848	2,964	61,887	1.190	0.034	2.24E-07	17	0.342	1.092	0.006	86.72
17	rs112114764	G/T	1.080	0.066	0.244	29,764	169,118	1.070	0.012	2.45E-08	14	1.30E-08	1.071	0.893	0
20	rs6130764	T/C	1.027	0.077	0.732	29,764	169,118	1.064	0.011	3.25E-08	14	3.51E-08	1.064	0.646	0

Note:
The OR is based on the first allele shown in the third column.
The sample size of each SNP in the original publications were also shown.
OR, odds ratio; SE, standard error; Ref., reference.

Table S2. Genotypic distributions of the 21 SNPs in our Han Chinese samples (1,146 BPD cases and 1,956 controls).

CHR	SNP	Position	Nearest Gene	A1	A2	TEST	Case	Control	2-tailed P-value
2	rs17183814	166152389	*SCN2A*	A	G	GENO	22/241/863	52/459/1445	0.174
3	rs2302417	52814256	*ITIH3*	A	T	GENO	173/515/414	322/934/669	0.292
3	rs3804640	107793709	*CD47*	G	A	GENO	26/236/804	45/505/1404	0.0759
4	rs11724116	162294038	*FSTL5*	T	C	GENO	18/273/837	36/484/1436	0.841
6	rs10455979	166995260	*RPS6KA2*	C	G	GENO	98/452/541	144/787/953	0.429
7	rs4236274	1896413	*MAD1L1*	A	G	GENO	260/545/309	369/934/574	0.0393
7	rs4332037	1950809	*MAD1L1*	T	C	GENO	19/224/892	12/355/1589	0.00918
9	rs12553324	23347865	*ELAVL2*	C	G	GENO	286/553/282	460/988/507	0.469
10	rs10821745	62136206	*ANK3*	G	T	GENO	63/394/634	111/701/1144	0.978
10	rs78089757	127112829	intergenic	A	G	GENO	0/58/1078	0/74/1868	0.0969
11	rs174576	61603510	*FADS1/2*	A	C	GENO	249/523/345	376/931/649	0.104
11	rs7122539	66662731	*RCE1*	A	G	GENO	282/526/320	496/981/479	0.0504
11	rs12575685	70517927	*SHANK2*	A	G	GENO	92/439/591	131/775/1050	0.302
11	rs12290811	79083620	*ODZ4*	A	T	GENO	3/133/990	7/208/1739	0.572
11	rs329674	133776948	*LOC646543*	A	G	GENO	48/341/742	82/609/1265	0.851
12	rs10744560	2387099	*CACNA1C*	T	C	GENO	3/129/999	4/200/1748	0.606
15	rs4447398	42904904	*HAUS2*	A	C	GENO	76/459/597	149/771/1028	0.603
16	rs11647445	9926966	*GRIN2A*	G	T	GENO	10/152/972	12/271/1667	0.625
17	rs76317718	8222777	*ARHGEF15*	T	G	GENO	96/432/598	153/769/1034	0.735
17	rs112114764	42201041	*HDAC5*	T	G	GENO	48/352/732	82/648/1200	0.361
20	rs6130764	43750410	*STK4*	C	T	GENO	17/284/823	34/493/1413	0.887

Table S3. Functional prediction of the SNPs in high LD with rs174576 using GWAVA dataset							
**SNP**	rs174528	rs174529	rs174530	rs108499	rs509360	rs174533	rs174534
**Chromosome**	11	11	11	11	11	11	11
**Position**	61,543,499	61,543,961	61,546,592	61,547,237	61,548,559	61,549,025	61,549,458
**Region score**	0.22	0.3	0.37	0.42	0.34	0.26	0.33
**TSS score**	0.26	0.2	0.19	0.24	0.16	0.13	0.09
**Unmatched score**	0.51	0.59	0.61	0.41	0.48	0.48	0.43
**Average GERP**	0.17893	-1.23477	-1.94495	-0.67709	-0.04432	0.83033	-0.66566
**Average DAF**	0.00118	0.00118	0.00044	0.00042	0.00099	0.00134	0.00069
**Average het**	0.00097	0.00096	0.00063	0.00061	0.00122	0.0017	0.00094
**GERP**	2.14	1.34	-3.64	3.32	-2.42	0.295	-6.02
**%GC**	68.3%	64.4%	69.3%	65.3%	59.4%	60.4%	45.5%
**TSS distance**	-1578	-1116	290	-446	-849	-383	48
**SS distance**	-26	54	34	-74	39	-17	159
**ACCEPTOR**	-	-	-	-	-	-	-
**ATF3**	-	-	-	-	-	-	-
**BATF**	-	-	-	-	-	-	-
**BCL11A**	-	-	-	-	-	-	-
**BCL3**	-	-	-	-	-	-	-
**BCLAF1**	-	-	-	-	-	-	-
**BDP1**	-	-	-	-	-	-	-
**BHLHE40**	-	-	-	-	-	-	-
**BRCA1**	-	-	-	-	-	-	-
**BRF1**	-	-	-	-	-	-	-
**BRF2**	-	-	-	-	-	-	-
**CCNT2**	-	-	-	-	-	-	-
**CDS**	-	-	-	-	-	-	-
**CEBPB**	-	-	-	-	-	-	-
**CHD2**	-	-	-	-	-	-	-
**CTBP2**	-	-	-	-	-	-	-
**CTCF**	-	1	-	-	-	-	-
**CTCFL**	-	-	-	-	-	-	-
**CTCF_REG**	-	5	-	-	-	-	-
**DNase**	5	2	2	-	5	2	1
**DONOR**	-	-	-	-	-	-	-
**E2F1**	-	-	-	-	-	-	-
**E2F4**	-	-	-	-	-	-	-
**E2F6**	-	-	-	-	-	-	-
**EBF1**	-	-	-	-	-	-	-
**EGR1**	-	-	-	-	-	-	-
**ELF1**	-	-	-	-	-	-	-
**ELK4**	-	-	-	-	-	-	-
**ENH**	-	-	-	-	-	-	-
**EP300**	-	-	-	-	-	-	-
**ERALPHAA**	-	-	-	-	-	-	-
**ESRRA**	-	-	-	-	-	-	-
**ETS1**	-	-	-	-	-	-	-
**EXON**	-	-	1	-	-	-	1
**Eralphaa**	-	-	-	-	-	-	-
**FAIRE**	2	-	-	-	1	-	-
**FAM48A**	-	-	-	-	-	-	-
**FOS**	-	-	-	-	-	-	-
**FOSL1**	-	-	-	-	-	-	-
**FOSL2**	-	-	-	-	-	-	-
**FOXA1**	-	-	-	-	-	-	-
**FOXA2**	-	-	-	-	-	-	-
**GABPA**	-	-	-	-	-	-	-
**GATA1**	-	-	-	-	-	-	-
**GATA2**	-	-	-	-	-	-	-

SNP	rs174528	rs174529	rs174530	rs108499	rs509360	rs174533	rs174534
GATA3	-	-	-	-	-	-	-
GTF2B	-	-	-	-	-	-	-
GTF2F1	-	-	-	-	-	-	-
GTF3C2	-	-	-	-	-	-	-
H2AFZ	-	-	-	-	-	-	-
H3K27ac	-	-	-	-	-	-	-
H3K27me3	1	1	1	-	-	-	-
H3K36me3	2	2	3	4	5	6	6
H3K4me1	3	3	1	-	2	2	2
H3K4me2	-	-	-	-	1	1	-
H3K4me3	-	-	-	-	-	-	-
H3K79me2	-	-	-	-	-	-	-
H3K9ac	2	2	1	1	1	1	1
H3K9me1	-	-	-	-	-	-	-
H3K9me3	-	-	-	-	-	-	-
H4K20me1	-	-	-	-	-	-	-
HDAC2	-	-	-	-	-	-	-
HDAC8	-	-	-	-	-	-	-
HEY1	-	-	-	-	-	-	-
HMGN3	-	-	-	-	-	-	-
HNF4A	-	-	-	-	-	-	-
HNF4G	-	-	-	-	-	-	-
HSF1	-	-	-	-	-	-	-
INTRON	1	1	1	1	1	1	1
IRF1	-	-	-	-	-	-	-
IRF3	-	-	-	-	-	-	-
IRF4	-	-	-	-	-	-	-
JUN	-	-	-	-	-	-	-
JUNB	-	-	-	-	-	-	-
JUND	-	-	-	-	-	-	-
KAT2A	-	-	-	-	-	-	-
MAFF	-	-	-	-	-	-	-
MAFK	-	-	-	-	-	-	-
MAX	-	-	-	-	-	-	-
MEF2A	-	-	-	-	-	-	-
MEF2_complex	-	-	-	-	-	-	-
MXI1	-	-	-	-	-	-	-
MYC	-	-	-	-	-	-	-
NANOG	-	-	-	-	-	-	-
NFE2	-	-	-	-	-	-	-
NFKB1	-	-	-	-	-	-	-
NFYA	-	-	-	-	-	-	-
NFYB	-	-	-	-	-	-	-
NR2C2	-	-	-	-	-	-	-
NR3C1	-	-	-	-	-	-	-
NR4A1	-	-	-	-	-	-	-
NRF1	-	-	-	-	-	-	-
PAX5	-	-	-	-	-	-	-
PBX3	-	-	-	-	-	-	-
POLR2A	-	-	-	-	1	-	-
POLR2A_elongating	-	-	-	-	-	-	-
POLR3A	-	-	-	-	-	-	-
POU2F2	-	-	-	-	-	-	-
POU5F1	-	-	-	-	-	-	-
PPARGC1A	-	-	-	-	-	-	-
PRDM1	-	-	-	-	-	-	-
RAD21	-	-	-	-	-	-	-
RDBP	-	-	-	-	-	-	-

SNP	rs174528	rs174529	rs174530	rs108499	rs509360	rs174533	rs174534
REP	-	-	-	-	-	1	-
REST	-	-	-	-	-	-	-
RFX5	-	-	-	-	-	-	-
RXRA	-	-	-	-	-	-	-
SETDB1	-	-	-	-	-	-	-
SIN3A	-	-	-	-	-	-	-
SIRT6	-	-	-	-	-	-	-
SIX5	-	-	-	-	-	-	-
SLC22A2	-	-	-	-	-	-	-
SMARCA4	-	-	-	-	-	-	-
SMARCB1	-	-	-	-	-	-	-
SMARCC1	-	-	-	-	-	-	-
SMARCC2	-	-	-	-	-	-	-
SMC3	-	-	-	-	-	-	-
SP1	-	-	-	-	-	-	-
SP2	-	-	-	-	-	-	-
SPI1	-	-	-	-	-	-	-
SREBF1	-	-	-	-	-	-	-
SREBF2	-	-	-	-	-	-	-
SRF	-	-	-	-	-	-	-
START	-	-	-	-	-	-	-
STAT1	-	-	-	-	-	-	-
STAT2	-	-	-	-	-	-	-
STAT3	-	-	-	-	-	-	-
STOP	-	-	-	-	-	-	-
SUZ12	-	-	-	-	-	-	-
TAF1	-	-	-	-	-	-	-
TAF7	-	-	-	-	-	-	-
TAL1	-	-	-	-	-	-	-
TBP	-	-	-	-	-	-	-
TCF12	-	-	-	-	-	-	-
TCF7L2	-	-	-	-	-	-	-
TFAP2A	-	-	-	-	-	-	-
TFAP2C	-	-	-	-	-	-	-
THAP1	-	-	-	-	-	-	-
TRAN	2	-	3	4	4	4	5
TRIM28	-	-	-	-	-	-	-
TSS	-	-	-	-	-	-	-
TSS_FLANK	-	-	-	-	-	-	-
USF1	-	-	-	-	-	-	2
USF2	-	-	-	-	-	-	-
UTR3	-	-	-	-	-	-	-
UTR5	-	-	-	-	-	-	-
WEAK_ENH	-	-	-	-	-	-	-
WRNIP1	-	-	-	-	-	-	-
XRCC4	-	-	-	-	-	-	-
YY1	-	-	-	-	-	-	-
ZBTB33	-	-	-	-	-	-	-
ZBTB7A	-	-	-	-	-	-	-
ZEB1	-	-	-	-	-	-	-
ZNF143	-	-	-	-	-	-	-
ZNF263	-	-	-	-	-	-	-
ZNF274	-	-	-	-	-	-	-
ZZZ3	-	-	-	-	-	-	-
bound_motifs	-	-	-	-	1	1	-
cpg_island	-	-	-	-	-	-	-
dnase_fps	-	-	-	-	-	-	-
in_cpg	-	-	-	1	-	-	-

SNP	rs174535	rs174536	rs174537	rs102275	rs102274	rs174538	rs174541
Chromosome	11	11	11	11	11	11	11
Position	61,551,356	61,551,927	61,552,680	61,557,803	61,557,826	61,560,081	61,565,908
Region score	0.3	0.41	0.37	0.25	0.26	0.8	0.3
TSS score	0.23	0.18	0.17	0.15	0.1	0.77	0.19
Unmatched score	0.42	0.45	0.38	0.62	0.54	0.98	0.35
Average GERP	2.6233	-0.67853	-1.56768	0.14267	0.11224	0.45534	-0.00782
Average DAF	0.00066	0.00071	0.00071	0.00124	0.00124	0.00042	0.00039
Average het	0.0005	0.00052	0.00051	0.00104	0.00105	0.00064	0.00055
GERP	1.33	-3.73	-4.16	2.94	-5.28	1.68	0.43
%GC	58.4%	60.4%	63.4%	49.5%	50.5%	65.3%	56.4%
TSS distance	1946	2517	3270	271	248	0	2495
SS distance	33	67	-589	-41	-18	53	1514
ACCEPTOR	-	-	-	-	-	-	-
ATF3	-	-	-	-	-	-	-
BATF	-	-	-	-	-	-	-
BCL11A	-	-	-	-	-	-	-
BCL3	-	-	-	-	-	-	-
BCLAF1	-	-	-	-	-	-	-
BDP1	-	-	-	-	-	-	-
BHLHE40	-	-	-	-	-	-	-
BRCA1	-	-	-	-	-	-	-
BRF1	-	-	-	-	-	-	-
BRF2	-	-	-	-	-	-	-
CCNT2	-	-	-	-	-	-	-
CDS	1	-	-	-	-	-	-
CEBPB	-	-	-	-	-	-	-
CHD2	-	-	-	-	-	-	-
CTBP2	-	-	-	-	-	-	-
CTCF	-	-	-	-	-	-	-
CTCFL	-	-	-	-	-	-	-
CTCF_REG	-	-	-	-	-	-	-
DNase	1	1	-	-	-	130	2
DONOR	-	-	-	-	-	-	-
E2F1	-	-	-	-	-	2	-
E2F4	-	-	-	-	-	-	-
E2F6	-	-	-	-	-	1	-
EBF1	-	-	-	-	-	-	-
EGR1	-	-	-	-	-	-	-
ELF1	-	-	-	-	-	3	-
ELK4	-	-	-	-	-	1	-
ENH	-	-	-	-	-	-	1
EP300	-	-	-	-	-	-	-
ERALPHAA	-	-	-	-	-	-	-
ESRRA	-	-	-	-	-	-	-
ETS1	-	-	-	-	-	-	-
EXON	1	1	1	1	1	1	-
Eralphaa	-	-	-	-	-	-	-
FAIRE	1	2	1	-	-	10	-
FAM48A	-	-	-	-	-	-	-
FOS	-	-	-	-	-	-	-
FOSL1	-	-	-	-	-	-	-
FOSL2	-	-	-	-	-	-	-
FOXA1	-	-	-	-	-	-	-
FOXA2	-	-	-	-	-	-	-
GABPA	-	-	-	-	-	5	-
GATA1	-	-	-	-	-	-	-
GATA2	-	-	-	-	-	-	-

SNP	rs174535	rs174536	rs174537	rs102275	rs102274	rs174538	rs174541
GATA3	-	-	-	-	-	-	-
GTF2B	-	-	-	-	-	-	-
GTF2F1	-	-	-	-	-	-	-
GTF3C2	-	-	-	-	-	-	-
H2AFZ	-	-	-	-	-	6	-
H3K27ac	2	2	-	3	4	12	5
H3K27me3	-	-	-	-	-	1	-
H3K36me3	10	18	26	27	27	-	9
H3K4me1	3	3	2	1	1	5	2
H3K4me2	-	-	-	1	1	13	1
H3K4me3	-	1	-	3	3	18	1
H3K79me2	-	-	-	6	6	6	5
H3K9ac	1	1	1	2	2	14	4
H3K9me1	-	-	-	-	-	-	-
H3K9me3	-	-	-	-	-	-	-
H4K20me1	-	-	-	-	-	-	-
HDAC2	-	-	-	-	-	-	-
HDAC8	-	-	-	-	-	-	-
HEY1	-	-	-	-	-	2	-
HMGN3	-	-	-	-	-	-	-
HNF4A	-	-	-	-	-	-	-
HNF4G	-	-	-	-	-	-	-
HSF1	-	-	-	-	-	-	-
INTRON	1	1	1	1	1	-	1
IRF1	-	-	-	-	-	-	-
IRF3	-	-	-	-	-	-	-
IRF4	-	-	-	-	-	-	-
JUN	-	-	-	-	-	-	-
JUNB	-	-	-	-	-	-	-
JUND	-	-	-	-	-	-	-
KAT2A	-	-	-	-	-	-	-
MAFF	-	-	-	-	-	-	-
MAFK	-	-	-	-	-	-	-
MAX	-	-	-	-	-	1	-
MEF2A	-	-	-	-	-	-	-
MEF2_complex	-	-	-	-	-	-	-
MXI1	-	-	-	-	-	-	-
MYC	-	-	-	-	-	-	-
NANOG	-	-	-	-	-	-	-
NFE2	-	-	-	-	-	-	-
NFKB1	-	-	-	-	-	3	-
NFYA	-	-	-	-	-	-	-
NFYB	-	-	-	-	-	-	-
NR2C2	-	-	-	-	-	-	-
NR3C1	-	-	-	-	-	-	-
NR4A1	-	-	-	-	-	-	-
NRF1	-	-	-	-	-	1	-
PAX5	-	-	-	-	-	1	-
PBX3	-	-	-	-	-	-	-
POLR2A	-	-	-	-	-	45	1
POLR2A_elongating	-	-	-	-	-	1	-
POLR3A	-	-	-	-	-	-	-
POU2F2	-	-	-	-	-	1	-
POU5F1	-	-	-	-	-	-	-
PPARGC1A	-	-	-	-	-	-	-
PRDM1	-	-	-	-	-	-	-
RAD21	-	-	-	-	-	-	-
RDBP	-	-	-	-	-	-	-

SNP	rs174535	rs174536	rs174537	rs102275	rs102274	rs174538	rs174541
REP	-	-	-	-	-	-	-
REST	-	-	-	-	-	-	-
RFX5	-	-	-	-	-	-	-
RXRA	-	-	-	-	-	-	-
SETDB1	-	-	-	-	-	-	-
SIN3A	-	-	-	-	-	1	-
SIRT6	-	-	-	-	-	-	-
SIX5	-	-	-	-	-	1	-
SLC22A2	-	-	-	-	-	1	-
SMARCA4	-	-	-	-	-	-	-
SMARCB1	-	-	-	-	-	-	-
SMARCC1	-	-	-	-	-	-	-
SMARCC2	-	-	-	-	-	-	-
SMC3	-	-	-	-	-	-	-
SP1	-	-	-	-	-	-	-
SP2	-	-	-	-	-	-	-
SPI1	-	-	-	-	-	3	-
SREBF1	-	-	-	-	-	-	-
SREBF2	-	-	-	-	-	-	-
SRF	-	-	-	-	-	2	-
START	-	-	-	-	-	-	-
STAT1	-	-	-	-	-	-	-
STAT2	-	-	-	-	-	-	-
STAT3	-	-	-	-	-	-	-
STOP	-	-	-	-	-	-	-
SUZ12	-	-	-	-	-	-	-
TAF1	-	-	-	-	-	8	-
TAF7	-	-	-	-	-	1	-
TAL1	-	-	-	-	-	-	-
TBP	-	-	-	-	-	5	-
TCF12	-	-	-	-	-	-	-
TCF7L2	-	-	-	-	-	-	-
TFAP2A	-	-	-	-	-	-	-
TFAP2C	-	-	-	-	-	-	-
THAP1	-	-	-	-	-	-	-
TRAN	6	6	6	6	6	-	5
TRIM28	-	-	-	-	-	-	-
TSS	-	-	-	-	-	6	-
TSS_FLANK	-	-	-	-	-	-	-
USF1	-	-	-	-	-	-	-
USF2	-	-	-	-	-	-	-
UTR3	-	-	-	-	-	-	-
UTR5	-	-	-	-	-	1	-
WEAK_ENH	-	-	-	-	-	-	-
WRNIP1	-	-	-	-	-	-	-
XRCC4	-	-	-	-	-	-	-
YY1	-	-	-	-	-	6	-
ZBTB33	-	-	-	-	-	-	-
ZBTB7A	-	-	-	-	-	-	-
ZEB1	-	-	-	-	-	-	-
ZNF143	-	-	-	-	-	1	-
ZNF263	-	-	-	-	-	-	-
ZNF274	-	-	-	-	-	-	-
ZZZ3	-	-	-	-	-	-	-
bound_motifs	-	-	-	-	-	1	-
cpg_island	-	-	-	-	-	1	-
dnase_fps	-	-	-	-	-	33	-
in_cpg	-	-	-	-	-	-	-

SNP	rs174544	rs174545	rs174546	rs174547	rs174548	rs174549	rs174550
Chromosome	11	11	11	11	11	11	11
Position	61,567,753	61,569,306	61,569,830	61,570,783	61,571,348	61,571,382	61,571,478
Region score	0.51	0.58	0.47	0.32	0.25	0.27	0.36
TSS score	0.35	0.35	0.36	0.13	0.14	0.16	0.22
Unmatched score	0.44	0.62	0.56	0.28	0.56	0.54	0.63
Average GERP	0.00461	0.0226	-0.35256	-0.79984	-1.1267	-1.02493	-0.12015
Average DAF	0.00034	0.00068	0.00068	0.00067	0.00202	0.00202	0.00202
Average het	0.00049	0.00046	0.00047	0.00045	0.00134	0.00134	0.00134
GERP	0.546	2.03	2.03	-1.35	-5.13	-5.23	0.64
%GC	61.4%	46.5%	54.5%	40.6%	51.5%	53.5%	49.5%
TSS distance	4340	2845	2321	1368	803	769	673
SS distance	-2181	-628	-104	-49	106	140	236
ACCEPTOR	-	-	-	-	-	-	-
ATF3	-	-	-	-	-	-	-
BATF	-	-	-	-	-	-	-
BCL11A	-	-	-	-	-	-	-
BCL3	-	-	-	-	-	-	-
BCLAF1	-	-	-	-	-	-	-
BDP1	-	-	-	-	-	-	-
BHLHE40	-	-	-	-	-	-	-
BRCA1	-	-	-	-	-	-	-
BRF1	-	-	-	-	-	-	-
BRF2	-	-	-	-	-	-	-
CCNT2	-	-	-	-	-	-	-
CDS	-	-	-	-	-	-	-
CEBPB	-	-	-	-	-	-	-
CHD2	-	-	-	-	-	-	-
CTBP2	-	-	-	-	-	-	-
CTCF	-	-	-	-	-	-	-
CTCFL	-	-	-	-	-	-	-
CTCF_REG	-	-	-	-	-	-	-
DNase	-	-	2	-	-	-	1
DONOR	-	-	-	-	-	-	-
E2F1	-	-	-	-	-	-	-
E2F4	-	-	-	-	-	-	-
E2F6	-	-	-	-	-	-	-
EBF1	-	-	-	-	-	-	-
EGR1	-	-	-	-	-	-	-
ELF1	-	-	-	-	-	-	-
ELK4	-	-	-	-	-	-	-
ENH	1	-	-	-	-	-	-
EP300	-	-	-	-	-	-	-
ERALPHAA	-	-	-	-	-	-	-
ESRRA	-	-	-	-	-	-	-
ETS1	-	-	-	-	-	-	-
EXON	1	1	1	-	1	1	1
Eralphaa	-	-	-	-	-	-	-
FAIRE	-	-	-	-	-	-	-
FAM48A	-	-	-	-	-	-	-
FOS	-	-	-	-	-	-	-
FOSL1	-	-	-	-	-	-	-
FOSL2	-	-	-	-	-	-	-
FOXA1	-	-	-	-	-	-	-
FOXA2	-	-	-	-	-	-	-
GABPA	-	-	-	-	-	-	-
GATA1	-	-	-	-	-	-	-
GATA2	-	-	-	-	-	-	-

SNP	rs174544	rs174545	rs174546	rs174547	rs174548	rs174549	rs174550
GATA3	-	-	-	-	-	-	-
GTF2B	-	-	-	-	-	-	-
GTF2F1	-	-	-	-	-	-	-
GTF3C2	-	-	-	-	-	-	-
H2AFZ	-	-	-	-	-	-	-
H3K27ac	3	1	2	2	2	2	2
H3K27me3	-	-	-	-	-	-	-
H3K36me3	15	14	25	26	25	25	25
H3K4me1	4	3	3	2	4	4	4
H3K4me2	1	1	1	1	-	-	-
H3K4me3	-	-	-	-	-	-	-
H3K79me2	4	4	3	3	2	2	2
H3K9ac	2	3	3	3	3	3	3
H3K9me1	-	-	-	-	-	-	-
H3K9me3	-	-	-	-	-	-	-
H4K20me1	-	-	-	-	-	-	-
HDAC2	-	-	-	-	-	-	-
HDAC8	-	-	-	-	-	-	-
HEY1	-	-	-	-	-	-	-
HMGN3	-	-	-	-	-	-	-
HNF4A	-	-	-	-	-	-	-
HNF4G	-	-	-	-	-	-	-
HSF1	-	-	-	-	-	-	-
INTRON	1	1	1	1	1	1	1
IRF1	-	-	-	-	-	-	-
IRF3	-	-	-	-	-	-	-
IRF4	-	-	-	-	-	-	-
JUN	-	-	-	-	-	-	-
JUNB	-	-	-	-	-	-	-
JUND	-	-	-	-	-	-	-
KAT2A	-	-	-	-	-	-	-
MAFF	-	-	-	-	-	-	-
MAFK	-	-	-	-	-	-	-
MAX	-	-	-	-	-	-	-
MEF2A	-	-	-	-	-	-	-
MEF2_complex	-	-	-	-	-	-	-
MXI1	-	-	-	-	-	-	-
MYC	-	-	-	-	-	-	-
NANOG	-	-	-	-	-	-	-
NFE2	-	-	-	-	-	-	-
NFKB1	-	-	-	-	-	-	-
NFYA	-	-	-	-	-	-	-
NFYB	-	-	-	-	-	-	-
NR2C2	-	-	-	-	-	-	-
NR3C1	-	-	-	-	-	-	-
NR4A1	-	-	-	-	-	-	-
NRF1	-	-	-	-	-	-	-
PAX5	-	-	-	-	-	-	-
PBX3	-	-	-	-	-	-	-
POLR2A	-	-	-	-	-	-	2
POLR2A_elongating	-	-	-	-	-	-	-
POLR3A	-	-	-	-	-	-	-
POU2F2	-	-	-	-	-	-	-
POU5F1	-	-	-	-	-	-	-
PPARGC1A	-	-	-	-	-	-	-
PRDM1	-	-	-	-	-	-	-
RAD21	-	-	-	-	-	-	-
RDBP	-	-	-	-	-	-	-

SNP	rs174544	rs174545	rs174546	rs174547	rs174548	rs174549	rs174550
REP	-	-	-	-	-	-	-
REST	-	-	-	-	-	-	-
RFX5	-	-	-	-	-	-	-
RXRA	-	-	-	-	-	-	-
SETDB1	-	-	-	-	-	-	-
SIN3A	-	-	-	-	-	-	-
SIRT6	-	-	-	-	-	-	-
SIX5	-	-	-	-	-	-	-
SLC22A2	-	-	-	-	-	-	-
SMARCA4	-	-	-	-	-	-	-
SMARCB1	-	-	-	-	-	-	-
SMARCC1	-	-	-	-	-	-	-
SMARCC2	-	-	-	-	-	-	-
SMC3	-	-	-	-	-	-	-
SP1	-	-	-	-	-	-	-
SP2	-	-	-	-	-	-	-
SPI1	-	-	-	-	-	-	-
SREBF1	-	-	-	-	-	-	-
SREBF2	-	-	-	-	-	-	-
SRF	-	-	-	-	-	-	-
START	-	-	-	-	-	-	-
STAT1	-	-	-	-	-	-	-
STAT2	-	-	-	-	-	-	-
STAT3	-	-	-	-	-	-	-
STOP	-	-	-	-	-	-	-
SUZ12	-	-	-	-	-	-	-
TAF1	-	-	-	-	-	-	-
TAF7	-	-	-	-	-	-	-
TAL1	-	-	-	-	-	-	-
TBP	-	-	-	-	-	-	-
TCF12	-	-	-	-	-	-	-
TCF7L2	-	-	-	-	-	-	-
TFAP2A	-	-	-	-	-	-	-
TFAP2C	-	-	-	-	-	-	-
THAP1	-	-	-	-	-	-	-
TRAN	5	6	5	6	6	6	6
TRIM28	-	-	-	-	-	-	-
TSS	-	-	-	-	-	-	-
TSS_FLANK	-	-	-	-	-	-	-
USF1	-	-	-	-	-	-	-
USF2	-	-	-	-	-	-	-
UTR3	1	1	1	-	-	-	-
UTR5	-	-	-	-	1	1	1
WEAK_ENH	-	-	-	-	-	-	-
WRNIP1	-	-	-	-	-	-	-
XRCC4	-	-	-	-	-	-	-
YY1	-	-	-	-	-	-	-
ZBTB33	-	-	-	-	-	-	-
ZBTB7A	-	-	-	-	-	-	-
ZEB1	-	-	-	-	-	-	-
ZNF143	-	-	-	-	-	-	-
ZNF263	-	-	-	-	-	-	-
ZNF274	-	-	-	-	-	-	-
ZZZ3	-	-	-	-	-	-	-
bound_motifs	-	-	-	-	-	-	-
cpg_island	-	-	-	-	-	-	-
dnase_fps	-	-	-	-	-	-	-
in_cpg	-	-	-	-	-	-	-

SNP	rs174553	rs7264355	rs174554	rs174555	rs174556	rs174559	rs174560
Chromosome	11	11	11	11	11	11	11
Position	61,575,158	61,579,427	61,579,463	61,579,760	61,580,635	61,581,656	61,581,764
Region score	0.33	0.08	0.14	0.19	0.45	0.27	0.2
TSS score	0.12	0.11	0.13	0.12	0.34	0.23	0.19
Unmatched score	0.25	0.36	0.27	0.48	0.45	0.68	0.79
Average GERP	-1.03263	0.0876	0.11761	-0.98534	-0.21083	0.04866	-0.67002
Average DAF	0.00069	0.00255	0.00255	0.00255	0.00032	0.00094	0.00095
Average het	0.00049	0.00122	0.00122	0.0012	0.00046	0.00088	0.00088
GERP	-0.144	-0.47	0.158	-7.33	2.81	0.122	1.52
%GC	53.5%	53.5%	51.5%	46.5%	50.5%	56.4%	49.5%
TSS distance	-1157	527	491	194	-679	464	356
SS distance	959	-23	10	-180	-77	42	150
ACCEPTOR	-	-	-	-	-	-	-
ATF3	-	-	-	-	-	-	-
BATF	-	-	-	-	-	-	-
BCL11A	-	-	-	-	-	-	-
BCL3	-	-	-	-	-	-	-
BCLAF1	-	-	-	-	-	-	-
BDP1	-	-	-	-	-	-	-
BHLHE40	-	-	-	-	-	-	-
BRCA1	-	-	-	-	-	-	-
BRF1	-	-	-	-	-	-	-
BRF2	-	-	-	-	-	-	-
CCNT2	-	-	-	-	-	-	-
CDS	-	1	-	-	-	-	-
CEBPB	-	-	-	-	2	-	-
CHD2	-	-	-	-	-	-	-
CTBP2	-	-	-	-	-	-	-
CTCF	-	-	-	-	-	-	-
CTCFL	-	-	-	-	-	-	-
CTCF_REG	-	-	-	-	-	-	-
DNase	-	-	-	-	-	-	1
DONOR	-	-	-	-	-	-	-
E2F1	-	-	-	-	-	-	-
E2F4	-	-	-	-	-	-	-
E2F6	-	-	-	-	-	-	-
EBF1	-	-	-	-	-	-	-
EGR1	-	-	-	-	-	-	-
ELF1	-	-	-	-	-	-	-
ELK4	-	-	-	-	-	-	-
ENH	-	-	-	-	-	-	1
EP300	-	-	-	-	-	-	-
ERALPHAA	-	-	-	-	-	-	-
ESRRA	-	-	-	-	-	-	-
ETS1	-	-	-	-	-	-	-
EXON	-	1	-	-	-	1	1
Eralphaa	-	-	-	-	-	-	-
FAIRE	-	-	-	-	-	1	-
FAM48A	-	-	-	-	-	-	-
FOS	-	-	-	-	-	-	-
FOSL1	-	-	-	-	-	-	-
FOSL2	-	-	-	-	-	-	-
FOXA1	-	-	-	-	-	-	-
FOXA2	-	-	-	-	-	-	-
GABPA	-	-	-	-	-	-	-
GATA1	-	-	-	-	-	-	-
GATA2	-	-	-	-	-	-	-

SNP	rs174553	rs7264355	rs174554	rs174555	rs174556	rs174559	rs174560
GATA3	-	-	-	-	-	-	-
GTF2B	-	-	-	-	-	-	-
GTF2F1	-	-	-	-	-	-	-
GTF3C2	-	-	-	-	-	-	-
H2AFZ	-	-	-	-	-	3	3
H3K27ac	-	-	-	1	1	10	10
H3K27me3	-	-	-	-	-	-	-
H3K36me3	17	27	27	27	26	5	5
H3K4me1	2	3	3	3	3	8	8
H3K4me2	-	1	1	2	2	9	12
H3K4me3	-	1	1	2	1	8	8
H3K79me2	-	2	2	3	3	4	4
H3K9ac	1	2	2	2	2	7	9
H3K9me1	-	-	-	-	-	-	-
H3K9me3	-	-	-	-	-	-	-
H4K20me1	-	-	-	-	-	-	-
HDAC2	-	-	-	-	-	-	-
HDAC8	-	-	-	-	-	-	-
HEY1	-	-	-	-	-	-	-
HMGN3	-	-	-	-	-	-	-
HNF4A	-	-	-	-	-	-	-
HNF4G	-	-	-	-	-	-	-
HSF1	-	-	-	-	-	-	-
INTRON	1	1	1	1	1	1	1
IRF1	-	-	-	-	-	-	-
IRF3	-	-	-	-	-	-	-
IRF4	-	-	-	-	-	-	-
JUN	-	-	-	-	-	-	-
JUNB	-	-	-	-	-	-	-
JUND	-	-	-	-	-	-	-
KAT2A	-	-	-	-	-	-	-
MAFF	-	-	-	-	-	-	-
MAFK	-	-	-	-	-	-	-
MAX	-	-	-	-	-	-	-
MEF2A	-	-	-	-	-	-	-
MEF2_complex	-	-	-	-	-	-	-
MXI1	-	-	-	-	-	-	-
MYC	-	-	-	-	-	-	-
NANOG	-	-	-	-	-	-	-
NFE2	-	-	-	-	-	-	-
NFKB1	-	-	-	-	-	-	-
NFYA	-	-	-	-	-	-	-
NFYB	-	-	-	-	-	-	-
NR2C2	-	-	-	-	-	-	-
NR3C1	-	-	-	-	-	-	-
NR4A1	-	-	-	-	-	-	-
NRF1	-	-	-	-	-	-	-
PAX5	-	-	-	-	-	-	-
PBX3	-	-	-	-	-	-	-
POLR2A	-	-	-	-	-	2	2
POLR2A_elongating	-	-	-	-	-	-	-
POLR3A	-	-	-	-	-	-	-
POU2F2	-	-	-	-	-	-	-
POU5F1	-	-	-	-	-	-	-
PPARGC1A	-	-	-	-	-	-	-
PRDM1	-	-	-	-	-	-	-
RAD21	-	-	-	-	-	-	-
RDBP	-	-	-	-	-	-	-

SNP	rs174553	rs7264355	rs174554	rs174555	rs174556	rs174559	rs174560
REP	-	-	-	-	-	-	-
REST	-	-	-	-	-	-	-
RFX5	-	-	-	-	-	-	-
RXRA	-	-	-	-	-	-	-
SETDB1	-	-	-	-	-	-	-
SIN3A	-	-	-	-	-	-	-
SIRT6	-	-	-	-	-	-	-
SIX5	-	-	-	-	-	-	-
SLC22A2	-	-	-	-	-	-	-
SMARCA4	-	-	-	-	-	-	-
SMARCB1	-	-	-	-	-	-	-
SMARCC1	-	-	-	-	-	-	-
SMARCC2	-	-	-	-	-	-	-
SMC3	-	-	-	-	-	-	-
SP1	-	-	-	-	-	-	-
SP2	-	-	-	-	-	-	-
SPI1	-	-	-	-	-	-	-
SREBF1	-	-	-	-	-	-	-
SREBF2	-	-	-	-	-	-	-
SRF	-	-	-	-	-	-	-
START	-	-	-	-	-	-	-
STAT1	-	-	-	-	-	-	-
STAT2	-	-	-	-	-	-	-
STAT3	-	-	-	-	-	-	-
STOP	-	-	-	-	-	-	-
SUZ12	-	-	-	-	-	-	-
TAF1	-	-	-	-	-	-	-
TAF7	-	-	-	-	-	-	-
TAL1	-	-	-	-	-	-	-
TBP	-	-	-	-	-	-	-
TCF12	-	-	-	-	-	-	-
TCF7L2	-	-	-	-	-	-	-
TFAP2A	-	-	-	-	-	-	-
TFAP2C	-	-	-	-	-	-	-
THAP1	-	-	-	-	-	-	-
TRAN	6	6	6	6	6	2	2
TRIM28	-	-	-	-	-	-	-
TSS	-	-	-	-	-	-	-
TSS_FLANK	-	-	-	-	-	-	-
USF1	-	-	-	-	-	-	-
USF2	-	-	-	-	-	-	-
UTR3	-	-	-	-	-	-	-
UTR5	-	-	-	-	-	-	-
WEAK_ENH	-	-	-	-	-	-	-
WRNIP1	-	-	-	-	-	-	-
XRCC4	-	-	-	-	-	-	-
YY1	-	-	-	-	-	-	-
ZBTB33	-	-	-	-	-	-	-
ZBTB7A	-	-	-	-	-	-	-
ZEB1	-	-	-	-	-	-	-
ZNF143	-	-	-	-	-	-	-
ZNF263	-	-	-	-	-	-	-
ZNF274	-	-	-	-	-	-	-
ZZZ3	-	-	-	-	-	-	-
bound_motifs	-	-	-	-	-	-	-
cpg_island	-	-	-	-	-	-	-
dnase_fps	-	-	-	-	-	-	-
in_cpg	-	-	-	-	-	-	-

SNP	rs174561	rs174562	rs174566	rs174568	rs99780	rs174570	rs1535
Chromosome	11	11	11	11	11	11	11
Position	61,582,708	61,585,144	61,592,362	61,593,816	61,596,633	61,597,212	61,597,972
Region score	0.53	0.36	0.16	0.35	0.23	0.45	0.32
TSS score	0.33	0.5	0.18	0.44	0.36	0.3	0.44
Unmatched score	0.95	0.88	0.17	0.85	0.92	0.77	0.72
Average GERP	-0.02572	-0.70901	0.12565	-0.61491	-0.55413	-0.76345	-0.76724
Average DAF	0.00075	0.00071	0.00292	0.00041	0.00061	0.00026	0.00087
Average het	0.00054	0.00052	0.0016	0.0006	0.00052	0.0004	0.00083
GERP	2.22	0.315	0.575	-0.992	-2.45	-1.9	-0.318
%GC	67.3%	50.5%	48.5%	67.3%	55.4%	48.5%	40.6%
TSS distance	3	-474	-2501	-1047	147	-421	-1181
SS distance	162	680	-1139	61	-37	539	1299
ACCEPTOR	-	-	-	-	-	-	-
ATF3	-	-	-	-	-	-	-
BATF	-	-	-	-	-	-	-
BCL11A	-	-	-	-	-	-	-
BCL3	-	-	-	-	-	-	-
BCLAF1	-	-	-	-	-	-	-
BDP1	-	-	-	-	-	-	-
BHLHE40	-	-	-	-	-	-	-
BRCA1	-	-	-	-	-	-	-
BRF1	-	-	-	-	-	-	-
BRF2	-	-	-	-	-	-	-
CCNT2	-	-	-	-	-	-	-
CDS	-	-	-	1	-	-	-
CEBPB	-	-	-	-	-	-	-
CHD2	-	-	-	-	-	-	-
CTBP2	-	-	-	-	-	-	-
CTCF	-	-	-	-	-	-	-
CTCFL	-	-	-	-	-	-	-
CTCF_REG	-	-	-	-	-	-	-
DNase	105	5	-	-	16	-	7
DONOR	-	-	-	-	-	-	-
E2F1	-	-	-	-	-	-	-
E2F4	-	-	-	-	-	-	-
E2F6	-	-	-	-	-	-	-
EBF1	-	-	-	-	-	-	-
EGR1	-	-	-	-	-	-	-
ELF1	-	-	-	-	-	-	-
ELK4	-	-	-	-	-	-	-
ENH	-	1	-	-	-	-	-
EP300	-	-	-	-	-	-	-
ERALPHAA	-	-	-	-	-	-	1
ESRRA	-	-	-	-	-	-	-
ETS1	-	-	-	-	-	-	-
EXON	1	-	-	1	-	-	-
Eralphaa	-	-	-	-	-	-	1
FAIRE	9	-	-	3	1	2	3
FAM48A	-	-	-	-	-	-	-
FOS	-	-	-	-	-	-	-
FOSL1	-	-	-	-	-	-	-
FOSL2	-	-	-	-	-	-	-
FOXA1	-	-	-	-	-	-	-
FOXA2	-	-	-	-	-	-	-
GABPA	-	-	-	-	-	-	-
GATA1	-	-	-	-	-	-	-
GATA2	-	-	-	-	-	-	-

SNP	rs174561	rs174562	rs174566	rs174568	rs99780	rs174570	rs1535
GATA3	1	-	-	-	-	-	-
GTF2B	-	-	-	-	-	-	-
GTF2F1	-	-	-	-	-	-	-
GTF3C2	-	-	-	-	-	-	-
H2AFZ	4	5	-	-	5	1	1
H3K27ac	11	9	-	3	10	8	4
H3K27me3	-	-	-	-	1	1	-
H3K36me3	2	-	-	1	1	2	2
H3K4me1	8	7	-	2	10	13	11
H3K4me2	13	13	-	2	11	11	11
H3K4me3	14	14	-	4	17	17	15
H3K79me2	4	3	-	1	3	3	3
H3K9ac	14	14	-	4	11	11	8
H3K9me1	-	-	-	-	-	-	-
H3K9me3	-	-	-	-	-	-	-
H4K20me1	-	-	-	-	2	3	3
HDAC2	-	-	-	-	-	-	-
HDAC8	-	-	-	-	-	-	-
HEY1	-	-	-	-	-	-	-
HMGN3	1	-	-	-	-	-	-
HNF4A	-	-	-	-	-	-	-
HNF4G	-	-	-	-	-	-	-
HSF1	-	-	-	-	-	-	-
INTRON	1	1	1	1	1	1	1
IRF1	1	-	-	-	-	-	-
IRF3	-	-	-	-	-	-	-
IRF4	-	-	-	-	-	-	-
JUN	-	-	-	-	-	-	-
JUNB	-	-	-	-	-	-	-
JUND	-	-	-	-	-	-	-
KAT2A	-	-	-	-	-	-	-
MAFF	-	-	-	-	-	-	-
MAFK	-	-	-	-	-	-	-
MAX	-	-	-	-	-	-	-
MEF2A	-	-	-	-	-	-	-
MEF2_complex	-	-	-	-	-	-	-
MXI1	1	-	-	-	-	-	-
MYC	-	-	-	-	-	-	-
NANOG	1	-	-	-	-	-	-
NFE2	-	-	-	-	-	-	-
NFKB1	-	-	-	-	-	-	-
NFYA	-	-	-	-	-	-	-
NFYB	1	-	-	-	-	-	-
NR2C2	-	-	-	-	-	-	-
NR3C1	-	-	-	-	-	-	-
NR4A1	-	-	-	-	-	-	-
NRF1	4	-	-	-	-	-	-
PAX5	1	-	-	-	-	-	-
PBX3	-	-	-	-	-	-	-
POLR2A	7	-	-	-	2	-	-
POLR2A_elongating	1	-	-	-	-	-	-
POLR3A	-	-	-	-	-	-	-
POU2F2	-	-	-	-	-	-	-
POU5F1	-	-	-	-	-	-	-
PPARGC1A	-	-	-	-	-	-	-
PRDM1	-	-	-	-	-	-	-
RAD21	-	-	-	-	-	-	-
RDBP	-	-	-	-	-	-	-

SNP	rs174561	rs174562	rs174566	rs174568	rs99780	rs174570	rs1535
REP	-	-	3	1	-	-	1
REST	-	-	-	-	-	-	-
RFX5	1	-	-	-	-	-	-
RXRA	-	-	-	-	-	-	-
SETDB1	-	-	-	-	-	-	-
SIN3A	-	-	-	-	-	-	-
SIRT6	-	-	-	-	-	-	-
SIX5	-	-	-	-	-	-	-
SLC22A2	-	-	-	-	-	-	-
SMARCA4	-	-	-	-	-	-	-
SMARCB1	-	-	-	-	-	-	-
SMARCC1	-	-	-	-	-	-	-
SMARCC2	-	-	-	-	-	-	-
SMC3	-	-	-	-	-	-	-
SP1	1	-	-	-	-	-	-
SP2	-	-	-	-	-	-	-
SPI1	-	-	-	-	-	-	-
SREBF1	2	-	-	-	-	-	-
SREBF2	-	-	-	-	-	-	-
SRF	-	-	-	-	-	-	-
START	-	-	-	-	-	-	-
STAT1	-	-	-	-	-	-	-
STAT2	-	-	-	-	-	-	-
STAT3	-	-	-	-	-	-	-
STOP	-	-	-	-	-	-	-
SUZ12	-	-	-	-	-	-	-
TAF1	-	-	-	-	-	-	-
TAF7	-	-	-	-	-	-	-
TAL1	-	-	-	-	-	-	-
TBP	-	-	-	-	-	-	-
TCF12	-	-	-	-	-	-	-
TCF7L2	-	-	-	-	-	-	-
TFAP2A	-	-	-	-	-	-	-
TFAP2C	-	-	-	-	-	-	-
THAP1	-	-	-	-	-	-	-
TRAN	-	-	3	4	1	1	-
TRIM28	-	-	-	-	-	-	-
TSS	6	2	-	-	4	2	1
TSS_FLANK	-	-	-	-	-	-	-
USF1	-	-	-	-	-	-	-
USF2	1	-	-	-	-	-	-
UTR3	-	-	-	1	-	-	-
UTR5	1	-	-	-	-	-	-
WEAK_ENH	-	-	-	-	-	-	-
WRNIP1	-	-	-	-	-	-	-
XRCC4	-	-	-	-	-	-	-
YY1	-	-	-	-	-	-	-
ZBTB33	-	-	-	-	-	-	-
ZBTB7A	-	-	-	-	-	-	-
ZEB1	-	-	-	-	-	-	-
ZNF143	-	-	-	-	-	-	-
ZNF263	-	-	-	-	-	-	-
ZNF274	-	-	-	-	-	-	-
ZZZ3	-	-	-	-	-	-	-
bound_motifs	-	-	-	-	1	-	1
cpg_island	1	-	-	-	1	-	-
dnase_fps	36	-	-	-	-	-	-
in_cpg	-	-	-	1	-	1	-

SNP	rs174574	rs174576	rs174577	rs174578	rs174580	rs174581	rs174583
Chromosome	11	11	11	11	11	11	11
Position	61,600,342	61,603,510	61,604,814	61,605,499	61,606,642	61,606,683	61,609,750
Region score	0.3	0.25	0.28	0.21	0.24	0.21	0.34
TSS score	0.3	0.19	0.08	0.14	0.09	0.07	0.25
Unmatched score	0.33	0.21	0.18	0.21	0.21	0.16	0.52
Average GERP	-0.92367	-0.42546	-0.79807	-0.61788	-0.77125	-0.52703	0.5894
Average DAF	0.0008	0.00171	0.00241	0.00161	0.00222	0.0024	0.00165
Average het	0.00094	0.00123	0.00151	0.00119	0.00124	0.00153	0.00078
GERP	-0.694	-2.97	-0.924	-4.83	0.0877	-2.78	-7.18
%GC	54.5%	36.6%	48.5%	63.4%	67.3%	66.3%	62.4%
TSS distance	-3551	-4258	-2954	-2269	-1126	-1085	1635
SS distance	3669	-1737	-433	136	-1161	-1120	1550
ACCEPTOR	-	-	-	-	-	-	-
ATF3	-	-	-	-	-	-	-
BATF	-	-	-	-	-	-	-
BCL11A	-	-	-	-	-	-	-
BCL3	-	-	-	-	-	-	-
BCLAF1	-	-	-	-	-	-	-
BDP1	-	-	-	-	-	-	-
BHLHE40	-	-	-	-	-	-	-
BRCA1	-	-	-	-	-	-	-
BRF1	-	-	-	-	-	-	-
BRF2	-	-	-	-	-	-	-
CCNT2	-	-	-	-	-	-	-
CDS	-	-	-	-	-	-	-
CEBPB	-	-	-	-	-	-	-
CHD2	-	-	-	-	-	-	-
CTBP2	-	-	-	-	-	-	-
CTCF	-	-	-	-	-	-	-
CTCFL	-	-	-	-	-	-	-
CTCF_REG	-	-	-	-	-	-	-
DNase	6	1	-	1	-	-	1
DONOR	-	-	-	-	-	-	-
E2F1	-	-	-	-	-	-	-
E2F4	-	-	-	-	-	-	-
E2F6	-	-	-	-	-	-	-
EBF1	-	-	-	-	-	-	-
EGR1	-	-	-	-	-	-	-
ELF1	-	-	-	-	-	-	-
ELK4	-	-	-	-	-	-	-
ENH	-	1	1	-	-	-	1
EP300	-	-	-	-	-	-	-
ERALPHAA	-	-	-	-	-	-	-
ESRRA	-	-	-	-	-	-	-
ETS1	-	-	-	-	-	-	-
EXON	-	-	-	-	-	-	-
Eralphaa	-	-	-	-	-	-	-
FAIRE	-	-	-	-	-	-	-
FAM48A	-	-	-	-	-	-	-
FOS	-	-	-	-	-	-	-
FOSL1	-	-	-	-	-	-	-
FOSL2	-	-	-	-	-	-	-
FOXA1	-	-	-	-	-	-	-
FOXA2	-	-	-	-	-	-	-
GABPA	-	-	-	-	-	-	-
GATA1	-	-	-	-	-	-	-
GATA2	-	-	-	-	-	-	-

SNP	rs174574	rs174576	rs174577	rs174578	rs174580	rs174581	rs174583
GATA3	-	-	-	-	-	-	-
GTF2B	-	-	-	-	-	-	-
GTF2F1	-	-	-	-	-	-	-
GTF3C2	-	-	-	-	-	-	-
H2AFZ	-	-	-	-	-	-	-
H3K27ac	4	4	3	1	1	1	1
H3K27me3	-	-	-	-	-	-	-
H3K36me3	10	2	16	18	18	17	23
H3K4me1	9	7	3	3	2	2	2
H3K4me2	5	4	-	-	-	-	1
H3K4me3	7	3	-	-	-	-	1
H3K79me2	2	2	2	2	-	-	-
H3K9ac	3	3	1	1	1	1	2
H3K9me1	-	-	-	-	-	-	-
H3K9me3	-	-	-	-	-	-	-
H4K20me1	3	-	-	-	-	-	-
HDAC2	-	-	-	-	-	-	-
HDAC8	-	-	-	-	-	-	-
HEY1	-	-	-	-	-	-	-
HMGN3	-	-	-	-	-	-	-
HNF4A	-	-	-	-	-	-	-
HNF4G	-	-	-	-	-	-	-
HSF1	-	-	-	-	-	-	-
INTRON	1	1	1	1	1	1	1
IRF1	-	-	-	-	-	-	-
IRF3	-	-	-	-	-	-	-
IRF4	-	-	-	-	-	-	-
JUN	-	-	-	-	-	-	-
JUNB	-	-	-	-	-	-	-
JUND	-	-	-	-	-	-	-
KAT2A	-	-	-	-	-	-	-
MAFF	-	-	-	-	-	-	-
MAFK	-	-	-	-	-	-	-
MAX	-	-	-	-	-	-	-
MEF2A	-	-	-	-	-	-	-
MEF2_complex	-	-	-	-	-	-	-
MXI1	-	-	-	-	-	-	-
MYC	-	-	-	-	-	-	-
NANOG	-	-	-	-	-	-	-
NFE2	-	-	-	-	-	-	-
NFKB1	-	-	-	-	-	-	-
NFYA	-	-	-	-	-	-	-
NFYB	-	-	-	-	-	-	-
NR2C2	-	-	-	-	-	-	-
NR3C1	-	-	-	-	-	-	-
NR4A1	-	-	-	-	-	-	-
NRF1	-	-	-	-	-	-	-
PAX5	-	-	-	-	-	-	-
PBX3	-	-	-	-	-	-	-
POLR2A	1	-	-	-	-	-	1
POLR2A_elongating	-	-	-	-	-	-	-
POLR3A	-	-	-	-	-	-	-
POU2F2	-	-	-	-	-	-	-
POU5F1	-	-	-	-	-	-	-
PPARGC1A	-	-	-	-	-	-	-
PRDM1	-	-	-	-	-	-	-
RAD21	-	-	-	-	-	-	-
RDBP	-	-	-	-	-	-	-

SNP	rs174574	rs174576	rs174577	rs174578	rs174580	rs174581	rs174583
REP	-	1	1	-	-	-	-
REST	-	-	-	-	-	-	-
RFX5	-	-	-	-	-	-	-
RXRA	-	-	-	-	-	-	-
SETDB1	-	-	-	-	-	-	-
SIN3A	-	-	-	-	-	-	-
SIRT6	-	-	-	-	-	-	-
SIX5	-	-	-	-	-	-	-
SLC22A2	-	-	-	-	-	-	-
SMARCA4	-	-	-	-	-	-	-
SMARCB1	-	-	-	-	-	-	-
SMARCC1	-	-	-	-	-	-	-
SMARCC2	-	-	-	-	-	-	-
SMC3	-	-	-	-	-	-	-
SP1	-	-	-	-	-	-	-
SP2	-	-	-	-	-	-	-
SPI1	-	-	-	-	-	-	-
SREBF1	-	-	-	-	-	-	-
SREBF2	-	-	-	-	-	-	-
SRF	-	-	-	-	-	-	-
START	-	-	-	-	-	-	-
STAT1	-	-	-	1	-	-	-
STAT2	-	-	-	1	-	-	-
STAT3	-	-	-	-	-	-	-
STOP	-	-	-	-	-	-	-
SUZ12	-	-	-	-	-	-	-
TAF1	-	-	-	-	-	-	-
TAF7	-	-	-	-	-	-	-
TAL1	-	-	-	-	-	-	-
TBP	-	-	-	-	-	-	-
TCF12	-	-	-	-	-	-	-
TCF7L2	-	-	-	-	-	-	-
TFAP2A	-	-	-	-	-	-	-
TFAP2C	-	-	-	-	-	-	-
THAP1	-	-	-	-	-	-	-
TRAN	4	2	3	4	5	5	5
TRIM28	-	-	-	-	-	-	-
TSS	-	-	-	-	-	-	-
TSS_FLANK	-	-	-	-	-	-	-
USF1	-	-	-	-	-	-	-
USF2	-	-	-	-	-	-	-
UTR3	-	-	-	-	-	-	-
UTR5	-	-	-	-	-	-	-
WEAK_ENH	-	-	-	-	-	-	-
WRNIP1	-	-	-	-	-	-	-
XRCC4	-	-	-	-	-	-	-
YY1	-	-	-	-	-	-	-
ZBTB33	-	-	-	-	-	-	-
ZBTB7A	-	-	-	-	-	-	-
ZEB1	-	-	-	-	-	-	-
ZNF143	-	-	-	-	-	-	-
ZNF263	-	-	-	-	-	-	-
ZNF274	-	-	-	-	-	-	-
ZZZ3	-	-	-	-	-	-	-
bound_motifs	-	-	-	-	-	-	-
cpg_island	-	-	-	-	-	-	-
dnase_fps	1	-	-	-	-	-	-
in_cpg	-	-	-	-	-	1	1

SNP	rs174584	rs174592	rs174594	rs7264355	rs174598	rs174599	rs174600
Chromosome	11	11	11	11	11	11	11
Position	61,610,750	61,618,608	61,619,829	61,620,274	61,621,194	61,621,556	61,622,227
Region score	0.31	0.15	0.26	0.24	0.28	0.33	0.24
TSS score	0.09	0.03	0.06	0.02	0.05	0.09	0.12
Unmatched score	0.12	0.13	0.11	0.05	0.18	0.27	0.42
Average GERP	0.05419	-0.27793	-0.22341	-0.06186	-1.53303	-0.20373	-0.91887
Average DAF	0.00076	0.00186	0.00083	0.00259	0.0045	0.00198	0.0026
Average het	0.00081	0.00105	0.00116	0.00237	0.00287	0.00133	0.00082
GERP	0.235	-0.897	0.546	-0.0767	-6.27	2.34	0.888
%GC	52.5%	40.6%	60.4%	39.6%	63.4%	67.3%	58.4%
TSS distance	2635	2971	-1829	-1384	-464	-102	567
SS distance	2550	1387	2608	3053	-3286	-2924	-2253
ACCEPTOR	-	-	-	-	-	-	-
ATF3	-	-	-	-	-	-	-
BATF	-	-	-	-	-	-	-
BCL11A	-	-	-	-	-	-	-
BCL3	-	-	-	-	-	-	-
BCLAF1	-	-	-	-	-	-	-
BDP1	-	-	-	-	-	-	-
BHLHE40	-	-	-	-	-	-	-
BRCA1	-	-	-	-	-	-	-
BRF1	-	-	-	-	-	-	-
BRF2	-	-	-	-	-	-	-
CCNT2	-	-	-	-	-	-	-
CDS	-	-	-	-	-	-	-
CEBPB	-	-	-	-	-	-	-
CHD2	-	-	-	-	-	-	-
CTBP2	-	-	-	-	-	-	-
CTCF	-	-	-	-	-	-	-
CTCFL	-	-	-	-	-	-	-
CTCF_REG	-	-	-	-	-	-	-
DNase	-	2	1	-	-	1	1
DONOR	-	-	-	-	-	-	-
E2F1	-	-	-	-	-	-	-
E2F4	-	-	-	-	-	-	-
E2F6	-	-	-	-	-	-	-
EBF1	-	-	-	-	-	-	-
EGR1	-	-	-	-	-	-	-
ELF1	-	-	-	-	-	-	-
ELK4	-	-	-	-	-	-	-
ENH	-	-	-	-	-	-	-
EP300	-	-	-	-	-	-	-
ERALPHAA	-	-	-	-	-	-	-
ESRRA	-	-	-	-	-	-	-
ETS1	-	-	-	-	-	-	-
EXON	-	-	-	-	-	-	1
Eralphaa	-	-	-	-	-	-	-
FAIRE	-	-	-	-	1	-	-
FAM48A	-	-	-	-	-	-	-
FOS	-	-	-	-	-	-	-
FOSL1	-	-	-	-	-	-	-
FOSL2	-	-	-	-	-	-	-
FOXA1	-	-	-	-	-	-	-
FOXA2	-	-	-	-	-	-	-
GABPA	-	-	-	-	-	-	-
GATA1	-	-	-	-	-	-	-
GATA2	-	-	-	-	-	-	-

SNP	rs174584	rs174592	rs174594	rs7264355	rs174598	rs174599	rs174600
GATA3	-	-	-	-	-	-	-
GTF2B	-	-	-	-	-	-	-
GTF2F1	-	-	-	-	-	-	-
GTF3C2	-	-	-	-	-	-	-
H2AFZ	-	-	-	-	-	-	-
H3K27ac	1	1	-	-	1	2	2
H3K27me3	-	-	-	-	-	-	-
H3K36me3	22	16	14	5	17	17	17
H3K4me1	2	2	2	1	2	2	2
H3K4me2	-	-	-	-	-	-	-
H3K4me3	-	-	-	-	-	-	-
H3K79me2	-	-	-	-	-	-	-
H3K9ac	2	1	1	-	1	1	1
H3K9me1	-	-	-	-	-	-	-
H3K9me3	-	-	-	-	-	-	-
H4K20me1	-	-	-	-	-	-	-
HDAC2	-	-	-	-	-	-	-
HDAC8	-	-	-	-	-	-	-
HEY1	-	-	-	-	-	-	-
HMGN3	-	-	-	-	-	-	-
HNF4A	-	-	-	-	-	-	-
HNF4G	-	-	-	-	-	-	-
HSF1	-	-	-	-	-	-	-
INTRON	1	1	1	1	1	1	1
IRF1	-	-	-	-	-	-	-
IRF3	-	-	-	-	-	-	-
IRF4	-	-	-	-	-	-	-
JUN	-	-	-	-	-	-	-
JUNB	-	-	-	-	-	-	-
JUND	-	-	-	-	-	-	-
KAT2A	-	-	-	-	-	-	-
MAFF	-	-	-	-	-	-	-
MAFK	-	-	-	-	-	-	-
MAX	-	-	-	-	-	-	-
MEF2A	-	-	-	-	-	-	-
MEF2_complex	-	-	-	-	-	-	-
MXI1	-	-	-	-	-	-	-
MYC	-	-	-	-	-	-	-
NANOG	-	-	-	-	-	-	-
NFE2	-	-	-	-	-	-	-
NFKB1	-	-	-	-	-	-	-
NFYA	-	-	-	-	-	-	-
NFYB	-	-	-	-	-	-	-
NR2C2	-	-	-	-	-	-	-
NR3C1	-	-	-	-	-	-	-
NR4A1	-	-	-	-	-	-	-
NRF1	-	-	-	-	-	-	-
PAX5	-	-	-	-	-	-	-
PBX3	-	-	-	-	-	-	-
POLR2A	-	-	-	-	-	-	2
POLR2A_elongating	-	-	-	-	-	-	-
POLR3A	-	-	-	-	-	-	-
POU2F2	-	-	-	-	-	-	-
POU5F1	-	-	-	-	-	-	-
PPARGC1A	-	-	-	-	-	-	-
PRDM1	-	-	-	-	-	-	-
RAD21	-	-	-	-	-	-	-
RDBP	-	-	-	-	-	-	-

SNP	rs174584	rs174592	rs174594	rs7264355	rs174598	rs174599	rs174600
REP	-	-	1	1	-	-	-
REST	-	-	-	-	-	-	-
RFX5	-	-	-	-	-	-	-
RXRA	-	-	-	-	-	-	-
SETDB1	-	-	-	-	-	-	-
SIN3A	-	-	-	-	-	-	-
SIRT6	-	-	-	-	-	-	-
SIX5	-	-	-	-	-	-	-
SLC22A2	-	-	-	-	-	-	-
SMARCA4	-	-	-	-	-	-	-
SMARCB1	-	-	-	-	-	-	-
SMARCC1	-	-	-	-	-	-	-
SMARCC2	-	-	-	-	-	-	-
SMC3	-	-	-	-	-	-	-
SP1	-	-	-	-	-	-	-
SP2	-	-	-	-	-	-	-
SPI1	-	-	-	-	-	-	-
SREBF1	-	-	-	-	-	-	-
SREBF2	-	-	-	-	-	-	-
SRF	-	-	-	-	-	-	-
START	-	-	-	-	-	-	-
STAT1	-	-	-	-	-	-	-
STAT2	-	-	-	-	-	-	-
STAT3	-	-	-	-	-	-	-
STOP	-	-	-	-	-	-	-
SUZ12	-	-	-	-	-	-	-
TAF1	-	-	-	-	-	-	-
TAF7	-	-	-	-	-	-	-
TAL1	-	-	-	-	-	-	-
TBP	-	-	-	-	-	-	-
TCF12	-	-	-	-	-	-	-
TCF7L2	-	-	-	-	-	-	-
TFAP2A	-	-	-	-	-	-	-
TFAP2C	-	-	-	-	-	-	-
THAP1	-	-	-	-	-	-	-
TRAN	5	6	4	5	4	5	5
TRIM28	-	-	-	-	-	-	-
TSS	-	-	-	-	-	-	-
TSS_FLANK	-	-	-	-	-	-	-
USF1	-	-	-	-	-	-	-
USF2	-	-	-	-	-	-	-
UTR3	-	-	-	-	-	-	-
UTR5	-	-	-	-	-	-	-
WEAK_ENH	-	-	-	-	-	-	-
WRNIP1	-	-	-	-	-	-	-
XRCC4	-	-	-	-	-	-	-
YY1	-	-	-	-	-	-	-
ZBTB33	-	-	-	-	-	-	-
ZBTB7A	-	-	-	-	-	-	-
ZEB1	-	-	-	-	-	-	-
ZNF143	-	-	-	-	-	-	-
ZNF263	-	-	-	-	-	-	-
ZNF274	-	-	-	-	-	-	-
ZZZ3	-	-	-	-	-	-	-
bound_motifs	-	-	-	-	1	-	-
cpg_island	-	-	-	-	-	-	-
dnase_fps	-	-	-	-	-	-	-
in_cpg	-	-	-	-	1	-	-

SNP	rs174601	rs97384
Chromosome	11	11
Position	61,623,140	61,624,181
Region score	0.3	0.16
TSS score	0.14	0.16
Unmatched score	0.42	0.37
Average GERP	-0.9696	-0.76994
Average DAF	0.00067	0.00125
Average het	0.00081	0.00134
GERP	-6.31	-2.12
%GC	60.4%	57.4%
TSS distance	1480	2521
SS distance	-1340	-299
ACCEPTOR	-	-
ATF3	-	-
BATF	-	-
BCL11A	-	-
BCL3	-	-
BCLAF1	-	-
BDP1	-	-
BHLHE40	-	-
BRCA1	-	-
BRF1	-	-
BRF2	-	-
CCNT2	-	-
CDS	-	-
CEBPB	-	-
CHD2	-	-
CTBP2	-	-
CTCF	-	-
CTCFL	-	-
CTCF_REG	-	-
DNase	-	-
DONOR	-	-
E2F1	-	-
E2F4	-	-
E2F6	-	-
EBF1	-	-
EGR1	-	-
ELF1	-	-
ELK4	-	-
ENH	-	-
EP300	-	-
ERALPHAA	-	-
ESRRA	-	-
ETS1	-	-
EXON	1	1
Eralphaa	-	-
FAIRE	1	2
FAM48A	-	-
FOS	-	-
FOSL1	-	-
FOSL2	-	-
FOXA1	-	-
FOXA2	-	-
GABPA	-	-
GATA1	-	-
GATA2	-	-

SNP	rs174601	rs97384
GATA3	-	-
GTF2B	-	-
GTF2F1	-	-
GTF3C2	-	-
H2AFZ	-	-
H3K27ac	1	-
H3K27me3	-	-
H3K36me3	16	17
H3K4me1	2	1
H3K4me2	-	-
H3K4me3	-	-
H3K79me2	-	-
H3K9ac	1	1
H3K9me1	-	-
H3K9me3	-	-
H4K20me1	-	-
HDAC2	-	-
HDAC8	-	-
HEY1	-	-
HMGN3	-	-
HNF4A	-	-
HNF4G	-	-
HSF1	-	-
INTRON	1	1
IRF1	-	-
IRF3	-	-
IRF4	-	-
JUN	-	-
JUNB	-	-
JUND	-	-
KAT2A	-	-
MAFF	-	-
MAFK	-	-
MAX	-	-
MEF2A	-	-
MEF2_complex	-	-
MXI1	-	-
MYC	-	-
NANOG	-	-
NFE2	-	-
NFKB1	-	-
NFYA	-	-
NFYB	-	-
NR2C2	-	-
NR3C1	-	-
NR4A1	-	-
NRF1	-	-
PAX5	-	-
PBX3	-	-
POLR2A	-	1
POLR2A_elongating	-	-
POLR3A	-	-
POU2F2	-	-
POU5F1	-	-
PPARGC1A	-	-
PRDM1	-	-
RAD21	-	-
RDBP	-	-

SNP	rs174601	rs97384
REP	1	-
REST	-	-
RFX5	-	-
RXRA	-	-
SETDB1	-	-
SIN3A	-	-
SIRT6	-	-
SIX5	-	-
SLC22A2	-	-
SMARCA4	-	-
SMARCB1	-	-
SMARCC1	-	-
SMARCC2	-	-
SMC3	-	-
SP1	-	-
SP2	-	-
SPI1	-	-
SREBF1	-	-
SREBF2	-	-
SRF	-	-
START	-	-
STAT1	-	-
STAT2	-	-
STAT3	-	-
STOP	-	-
SUZ12	-	-
TAF1	-	-
TAF7	-	-
TAL1	-	-
TBP	-	-
TCF12	-	-
TCF7L2	-	-
TFAP2A	-	-
TFAP2C	-	-
THAP1	-	-
TRAN	4	4
TRIM28	-	-
TSS	-	-
TSS_FLANK	-	-
USF1	-	-
USF2	-	-
UTR3	-	-
UTR5	-	-
WEAK_ENH	-	-
WRNIP1	-	-
XRCC4	-	-
YY1	-	-
ZBTB33	-	-
ZBTB7A	-	-
ZEB1	-	-
ZNF143	-	-
ZNF263	-	-
ZNF274	-	-
ZZZ3	-	-
bound_motifs	-	-
cpg_island	-	-
dnase_fps	-	-
in_cpg	-	-

Table S4. Association of rs174576 with nearby gene expression in Brain xQTL dataset.

SNP	SNPc	SNPpos	featureName	featur	featurePositic	SpearmanRh	pValue
rs174576	11	61603510	CCDC86	11	60617796	0.10761057	0.016727
rs174576	11	61603510	PTGDR2	11	60621196	0.00937032	0.83543
rs174576	11	61603510	PRPF19	11	60670345	-0.0271187	0.547623
rs174576	11	61603510	TMEM109	11	60690216	0.02598794	0.56445
rs174576	11	61603510	TMEM132A	11	60704249	0.03245677	0.471679
rs174576	11	61603510	SLC15A3	11	60718625	-0.0001759	0.996888
rs174576	11	61603510	VPS37C	11	60905668	0.07971639	0.076708
rs174576	11	61603510	VWCE	11	61053735	0.00086987	0.984614
rs174576	11	61603510	DDB1	11	61100441	0.00978811	0.828204
rs174576	11	61603510	DAK	11	61113329	0.01122774	0.803419
rs174576	11	61603510	CYB561A3	11	61124740	0.01509886	0.737808
rs174576	11	61603510	TMEM138	11	61136244	-0.0389197	0.388045
rs174576	11	61603510	TMEM216	11	61161356	0.01189519	0.791993
rs174576	11	61603510	CPSF7	11	61188867	-0.0211698	0.638795
rs174576	11	61603510	SDHAF2	11	61213417	-0.0422312	0.348925
rs174576	11	61603510	PPP1R32	11	61258286	-0.0125515	0.780801
rs174576	11	61603510	LRRC10B	11	61277347	0.02547579	0.572152
rs174576	11	61603510	SYT7	11	61348293	-0.1005008	0.0255
rs174576	11	61603510	DAGLA	11	61511004	0.06900632	0.125601
rs174576	11	61603510	AP002380.1	11	61534985	-0.0371227	0.410345
rs174576	11	61603510	MYRF	11	61551323	-0.1065796	0.017807
**rs174576**	**11**	**61603510**	**TMEM258**	**11**	**61557569**	**-0.322616**	**1.99E-13**
rs174576	11	61603510	FEN1	11	61564397	0.02951914	0.512741
**rs174576**	**11**	**61603510**	**FADS1**	**11**	**61581811**	**0.32852334**	**6.77E-14**
rs174576	11	61603510	FADS2	11	61632624	0.02031105	0.652466
rs174576	11	61603510	FADS3	11	61657685	-0.1119994	0.012744
rs174576	11	61603510	RAB3IL1	11	61687665	-0.0334547	0.458153
rs174576	11	61603510	BEST1	11	61729729	-0.0084106	0.85208
rs174576	11	61603510	FTH1	11	61732892	0.07827048	0.082227
rs174576	11	61603510	INCENP	11	61920145	0.02385279	0.596884
rs174576	11	61603510	ASRGL1	11	62159551	0.03690153	0.413141
rs174576	11	61603510	AHNAK	11	62303711	-0.0312475	0.488359
rs174576	11	61603510	EEF1G	11	62338474	0.02784405	0.53696
rs174576	11	61603510	TUT1	11	62348964	0.03791928	0.400367
rs174576	11	61603510	MTA2	11	62365761	-0.0468323	0.298882
rs174576	11	61603510	EML3	11	62379004	0.03023333	0.50259
rs174576	11	61603510	ROM1	11	62381730	-0.0077876	0.862924
rs174576	11	61603510	B3GAT3	11	62389420	0.05523804	0.220367
rs174576	11	61603510	GANAB	11	62402368	0.03704176	0.411367
rs174576	11	61603510	INTS5	11	62414320	0.01370964	0.761163
rs174576	11	61603510	C11orf98	11	62437504	0.02475963	0.583005
rs174576	11	61603510	UQCC3	11	62439584	-0.0081654	0.856345
rs174576	11	61603510	UBXN1	11	62445916	-0.0459113	0.308497
rs174576	11	61603510	RP11-831H9.1	11	62457747	0.02958276	0.511832
rs174576	11	61603510	BSCL2	11	62474668	0.02226496	0.621538
rs174576	11	61603510	GNG3	11	62476276	0.07766802	0.084619
rs174576	11	61603510	HNRNPUL2	11	62490336	0.01393308	0.757391
rs174576	11	61603510	TTC9C	11	62507448	0.05301661	0.239517
rs174576	11	61603510	ZBTB3	11	62521137	-0.0006525	0.988458
rs174576	11	61603510	POLR2G	11	62533966	-0.0193672	0.667626
rs174576	11	61603510	TAF6L	11	62549585	-2.13E-06	0.999962
rs174576	11	61603510	TMEM179B	11	62557179	0.01506211	0.738423

rs174576	11	61603510	TMEM223	11	62557443	-6.30E-05	0.998886
rs174576	11	61603510	NXF1	11	62571022	-0.0063242	0.888495
rs174576	11	61603510	STX5	11	62595049	0.00632659	0.888454

Table S5. Association of rs174576 with nearby gene expression in the frontal cortex of GTEx dataset.

Gene Symbol	Gencode ID	NES	T-statistic	P-Value
SYT7	ENSG00000011347.5	-0.027	-0.62	0.54
DAGLA	ENSG00000134780.5	-0.071	-1.8	0.071
MYRF	ENSG00000124920.9	-0.06	-0.94	0.35
FEN1	ENSG00000168496.3	0.011	0.2	0.84
TMEM258	ENSG00000134825.9	0.085	1.2	0.22
FADS1	ENSG00000149485.12	-0.26	-4.5	0.000016
FADS2	ENSG00000134824.9	0.073	0.98	0.33
FADS3	ENSG00000221968.4	0.0038	0.07	0.94
RAB3IL1	ENSG00000167994.7	-0.0026	-0.042	0.97
BEST1	ENSG00000167995.11	-0.06	-0.89	0.38
FTH1	ENSG00000167996.11	-0.051	-0.52	0.61
INCENP	ENSG00000149503.8	0.13	2.5	0.014

Table S6. Association of the 21 SNPs with major depressive disorder in Han Chinese using data from the published CONVERGE MDD GWAS.

CHR	SNP	POS	Nearest Gene	Allele	BPD analysis		CONVERGE MDD GWAS	
					OR	2-tailed P-value	OR	2-tailed P-value
2	rs17183814	166152389	*SCN2A*	G/A	1.142	2.02E-09	1.116	**0.00875**
3	rs2302417	52814256	*ITIH3*	T/A	1.076	6.59E-11	1.022	0.561
3	rs3804640	107793709	*CD47*	A/G	1.065	1.99E-08	1.032	0.415
4	rs11724116	162294038	*FSTL5*	C/T	1.088	2.37E-08	0.925	0.0644
6	rs10455979	166995260	*RPS6KA2*	G/C	1.064	4.31E-08	1.010	0.626
7	rs4236274	1896413	*MAD1L1*	A/G	1.149	8.49E-12	1.003	0.859
7	rs4332037	1950809	*MAD1L1*	T/C	1.170	1.91E-09	1.062	0.236
9	rs12553324	23347865	*ELAVL2*	G/C	1.120	5.87E-09	1.026	0.259
10	rs10821745	62136206	*ANK3*	G/T	1.145	6.76E-09	1.048	0.189
10	rs78089757	127112829	intergenic	A/G	1.410	3.99E-07	0.844	0.0842
11	rs174576	61603510	*FADS1/2*	A/C	1.130	1.34E-10	1.019	0.614
11	rs7122539	66662731	*RCE1*	G/A	1.067	3.77E-08	1.016	0.465
11	rs12575685	70517927	*SHANK2*	A/G	1.073	7.71E-09	1.095	**0.00558**
11	rs12290811	79083620	*ODZ4*	A/T	1.190	7.81E-11	1.019	0.754
11	rs329674	133776948	*LOC646543*	A/G	1.220	9.59E-08	0.927	0.0377
12	rs10744560	2387099	*CACNA1C*	T/C	1.076	3.62E-10	1.005	0.916
15	rs4447398	42904904	*HAUS2*	A/C	1.099	9.37E-09	1.011	0.890
16	rs11647445	9926966	*GRIN2A*	G/T	1.079	1.08E-10	1.028	0.958
17	rs76317718	8222777	*ARHGEF15*	G/T	1.190	2.24E-07	1.009	0.803
17	rs112114764	42201041	*HDAC5*	G/T	1.071	2.45E-08	1.005	0.867
20	rs6130764	43750410	*STK4*	T/C	1.064	3.25E-08	1.029	0.566
